# Discontinuous Galerkin isogeometric analysis for segmentations generating overlapping regions

**DOI:** 10.1080/00036811.2019.1698724

**Published:** 2019-12-06

**Authors:** Christoph Hofer, Ioannis Toulopoulos

**Affiliations:** a Institute of Computational Mathematics, Johannes Kepler University (JKU), Linz, Austria; b Johann Radon Institute for Computational and Applied Mathematics (RICAM), Austrian Academy of Sciences, Linz, Austria; c AC^2^T Research GmbH, Austrian Excellence Center for Tribology, Wiener Neustadt, Austria

**Keywords:** Elliptic diffusion problems, heterogeneous diffusion coefficients, isogeometric analysis, non-matching parametrized interfaces, overlapping patches, discontinuous Galerkin methods, consistency error, 65M12, 65M15

## Abstract

In the Isogeometric Analysis (IGA) framework, the computational domain has very often a multipatch representation. The multipatch domain can be obtained by a volume segmentation of a boundary represented domain, e.g. provided by a Computer Aided Design model. Typically, small gaps and overlapping regions can appear at the patch interfaces of such multipatch representations. In the current work, we consider multipatch representations having only small overlapping regions between the patches. We develop a Discontinuous Galerkin (DG)-IGA method that can be immediately applied to these representations. Our method appropriately connects the fluxes of the one face of the overlapping region with the flux of the opposite face. We provide a theoretical justification of our approach by splitting the whole error into two components: the first is related to the incorrect representation of the patches (consistency error) and the second to the approximation properties of the IGA space. We show bounds for both components of the error. We verify the theoretical error estimates in a series of numerical examples.

## 1. Introduction

Isogeometric Analysis (IGA) has been introduced in [1] as a new methodology for solving numerically Partial Differential Equations (PDE). The key idea of the IGA concept is to use the superior finite dimensional spaces, which are used in Computer Aided Design (CAD), e.g. B-splines, NURBS, for both the exact representation of the computational domain Ω and discretizing the PDE problem. Since this work, many applications of the IGA methodology in several fields have been discussed in several papers, see, e.g. the monograph [2] and the references therein, as well as the survey paper [3]. From a computational point of view, we can say that the numerical algorithm for constructing the B-spline (or NURBS) basis functions is quite simple. This helps to produce high-order approximate solutions. From the theoretical point of view, the fundamental approximation properties of the B-spline spaces on a reference domain are discussed in [4]. The approximation properties of the mapped B-spline (or NURBS) spaces are discussed in several papers, see e.g. [3,5–7].

**Figure 1.**
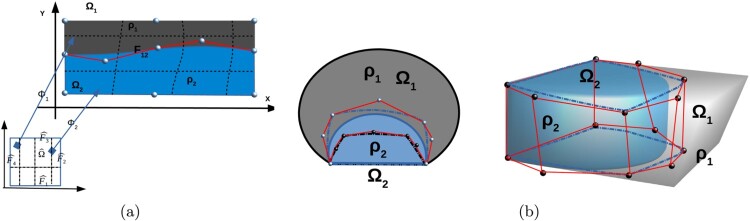
(a) A conforming multipatch representation of Ω, (b) the inaccurate control points and the non-conforming multipatch representation of Ω.

Let us consider a complex domain Ω where its boundary is prescribed by CAD models. The CAD models can not be directly used in IGA in order to discretize the PDE problems. We need to create volumetric patch parametrizations from the CAD models. The boundary represented domain is first segmented into a collection of suitable blocks and consequently, a parametrization procedure is applied to each block. This produces the volumetric multipatch representation $\overset{N}{\underset{i=1}{⋃}}\bar{\Omega_{i}}$ of $\bar{\Omega }$ suitable for IGA. Several segmentation algorithms and associated parametrization procedures have been discussed in the literature, see, e.g. [8–12]. Furthermore, we refer to [13–16] for different approaches for constructing IGA planar parametrizations without utilizing segmentation algorithms, and to [17, 18] for constructing parametrizations using Bézier triangular meshes. We mention the segmentation approach presented in [19,20], from which, we have been motivated to present the current work. The main idea is to split the given boundary represented domain, using a spline curve (or face in 3d case) with the following properties: (i) must have the end points on the boundary and the tangents to be specified, (ii) the curve is reasonably regular and does not intersect the boundary of the domain, (iii) the curve cuts the domain into new subdomains with good shapes. Consequently, tensor product B-spline spaces are fitted in the collection of the subdomains for defining the tensor product B-spline surfaces or volumes [14]. Note that the previous consideration also concerns CAD models that are connected along a non-matching interface. It is important to obtain a curve that splits Ω into new simple domains with good shapes being suitable for IGA. During the computation of the multipatch representation, errors can occur when defining the corresponding control points, see [10,14,19]. A consequence of this is non-conforming parametrizations of the patches in the sense that the images of the patch interfaces under the parametrizations are not identical. This, in turn, leads to the existence of gap and/or overlapping regions between the adjoining patches, see a schematic illustration in Figure 1(b).

This paper considers the case where there are only overlapping regions between the patches. If we apply an IGA methodology to this multipatch representation, a direct consequence is that the whole discretization error will include two (main) parts: the first naturally comes from the approximation properties of the B-spline spaces (for the purposes of this work we use B-spline spaces) and, the second comes from the geometric error. The later is due to the incorrect parametrization of the patch interfaces. Furthermore, the geometric error can be characterized as a consistency error, which consists of two error components. The first error component is related to the approximation of the jumps of the flux of the solution on the non-matching interfaces. The second component is related to the existence of more than one numerical solution in the overlapping regions.

The contribution of this paper is to develop a DG-IGA method which can be applied on volumetric patch representations with non-matching interface parametrizations. We present our methodology for discretizing the following elliptic Dirichlet boundary value problem

(1) $$-div(\rho \nabla u)=f\;in\;\Omega \;\text{and}\;u:=u_{D}=0\;on\;\partial \Omega ,$$

where the diffusion coefficient ρ(x) can be discontinuous across a smooth internal interface. We derive bounds for the two main parts of the whole error. In our analysis, we derive separate bounds for the two components of the geometric error. To the best of our knowledge, we believe that this is a new area of analysis to be investigated. Our current work is the first step in the analysis, where we are developing our methodology for the numerical solution of the simple stationary diffusion problem (1). Our intention for future works is to extend the current methodology to more complicated time dependent problems, where the interface can move with time, cf. [21].

Due to the non-matching interior patch interfaces, a direct application of the classical DG numerical fluxes proposed in the literature, see e.g. [6,22], is not possible, as these fluxes are only applicable for matching interface parametrizations. In our recent papers, [23,24], we developed DG-IGA schemes for multipatch unions that include only gap regions. In particular, we considered the PDE model given in (1) and we denoted by $d_{g}$ the maximum distance between the diametrically opposite points located on the gap boundary. We applied Taylor expansions using the diametrically opposite points of the gap, in order to give estimates for the jumps of the solution with respect to $d_{g}$. Finally, we used the same Taylor expansions in the DG-IGA scheme for constructing suitable DG numerical fluxes across the gap boundary that help on the weakly coupling of the local patch-wise discrete problems. We developed a discretization error analysis and showed a priori estimates in the DG-norm, expressed in terms of the mesh size and the gap width, i.e. $O(h^{r})+O(d_{g})$, where *r* depends on the B-spline degree *p* and the regularity of the solution. In [23,24], we have shown that, if $d_{g}=O(h^{p+1/2})$, the proposed DG-IGA scheme has optimal approximation properties.

In this paper, we extend the previous work to multipatch unions with overlapping regions. In the analysis presented in [23,24], the whole geometric error does not include the component coming from the coexistence of different IGA solutions in the overlapping regions. Here, the new approach is to introduce local (patch-wise) auxiliary variational problems, which are compatible with the overlapping nature of the multipatch representation of Ω. We denote the solutions of the new variational problems by $u^{*}$. These problems are not consistent, in the sense that the original solution *u* of (1) does not satisfy them. Following the IGA concept, the B-spline spaces used for the parametrization of the patches are also used for discretizing the local auxiliary problems. We denote by $u_{h}^{*}$ the produced IGA solutions. Under some regularity assumptions on $u^{*}$, we can expect (see Section 3) that the IGA solution $u_{h}^{*}$ has optimal approximation properties associated with $u^{*}$. However, we can not directly infer that $u_{h}^{*}$ can approximate in an optimal way the solution *u* of the original problem. In our analysis, we provide an estimate for the consistency error $u-u^{*}$ and consequently using the triangle inequality $∥u-u_{h}^{*}{∥}_{DG}\leq ∥u^{*}-u_{h}^{*}{∥}_{DG}+∥u-u^{*}{∥}_{DG}$, we can derive an estimate for the error between the exact solution *u* and the IGA solution $u_{h}^{*}$. The mesh-dependent norm $∥\cdot {∥}_{DG}$ is defined in Section 2. We give error estimates for both terms $∥u^{*}-u_{h}^{*}{∥}_{DG}$ and $∥u-u^{*}{∥}_{DG}$ expressed in terms of the mesh size *h* and the quantity $d_{o}$, which is introduced in our analysis in order to quantify the width of the overlapping regions. In particular, we show that under appropriate assumptions on the data and for the case where $d_{o}$ is of order $h^{\lambda },\lambda \geq p+\frac{1}{2}$, the proposed DG-IGA scheme has optimal convergence properties. This convergence result is similar to the result in [23,24].

In another work, which is under preparation, we apply the same approach to solve problems on multipatch partitions, which can include gap and overlapping regions. We present numerical solutions in multipatch unions with more complicated gaps and overlapping regions. We also provide details related to the implementation of the proposed DG-IGA scheme. In the same work, we also discuss issues related to the construction of domain-decomposition methods on these multipatch representations and provide several numerical tests for evaluating their performance. The first results in this direction can be found in [25].

We note that IGA multipatch representations with non-matching interfaces meshes, overlapping regions and trimmed patches have been considered in many publications. For the communication of the discrete patch-wise problems, several Nitsche's type coupling methods involving normal flux terms have been applied across the interfaces, see e.g. [22,26–28] and references therein. We mention also that in [29], DG-IGA methods have been presented to discretize Laplace problems on multipatch unions with large overlapping regions. The proposed strategy follows the additive Schwartz methodology. To the knowledge of the authors, there are no works that analytically discuss estimates for the error, which is caused by the incorrect representation of the shape of the patches. The purpose of this work is to present such an error analysis.

The structure of the paper is as follows: Section 2 presents the PDE model, briefly reviews the B-spline spaces and describes the case of having non-matching parametrized interfaces with overlapping regions. Section 3, presents in detail the perturbation problems, the bounds for the consistency error, the proposed DG-IGA scheme and the error analysis. Section 4, includes several numerical examples that confirm the theoretical estimates. The paper closes with the Conclusions.

## 2. The model problem

### 2.1. Preliminaries

Let Ω be a bounded Lipschitz domain in $R^{d},\;d=2,3$, and let $\alpha =(\alpha_{1},\ldots ,\alpha_{d})$ be a multi-index of non-negative integers $\alpha_{1},\ldots ,\alpha_{d}$ with degree $|\alpha |=\sum_{j=1}^{d}\alpha_{j}$. For any α, we define the differential operator $D^{\alpha }=D_{1}^{\alpha_{1}}\ldots D_{d}^{\alpha_{d}}$, with $D_{j}=\partial /\partial x_{j}$, j=1,…,d, and $D^{\left(0,\ldots ,0\right)}\varphi =\varphi$. For a non-negative integer *m*, let $C^{m}(\Omega )$ denote the space of all functions φ:Ω→R, whose partial derivatives $D^{\alpha }\varphi$ of all orders |α|≤m are continuous in Ω. Let ℓ be a non-negative integer. As usual, $L^{2}(\Omega )$ denotes the Sobolev space for which $\int_{\Omega }|\varphi (x){|}^{2}\;dx<\infty$, endowed with the norm $∥\varphi {∥}_{L^{2}(\Omega )}=(\int_{\Omega }|\varphi (x){|}^{2}\;dx{)}^{1/2}$, and $L^{\infty }(\Omega )$ denotes the functions that are essentially bounded. Also

$$H^{\ell }(\Omega )=\{\varphi \in L^{2}(\Omega ):D^{\alpha }\varphi \in L^{2}(\Omega ),\;for\;all\;|\alpha |\leq \ell \},$$

denote the standard Sobolev spaces endowed with the following norms

$$∥\varphi {∥}_{H^{\ell }(\Omega )}={\left(\sum_{0\leq |\alpha |\leq \ell }∥D^{\alpha }\varphi {∥}_{L^{2}(\Omega )}^{2}\right)}^{1/2}.$$

We identify $L^{2}$ and $H^{0}$ and also define the subspace $H_{0}^{1}(\Omega )$ and $H_{\Gamma }^{1}(\Omega )$ of $H^{1}(\Omega )$

$$H_{0}^{1}(\Omega )=\{\varphi \in H^{1}(\Omega ):\varphi =0\;on\;\partial \Omega \},\;H_{\Gamma }^{1}(\Omega )=\{\varphi \in H^{1}(\Omega ):\varphi =0\;on\;\Gamma \subset \partial \Omega ,\;|\Gamma |>0.\}.$$

We recall Hölder's and Young's inequalities

(2) $$\left|\int_{\Omega }\varphi_{1}\varphi_{2}\;dx\right|\leq ∥\varphi_{1}{∥}_{L^{2}(\Omega )}∥\varphi_{2}{∥}_{L^{2}(\Omega )}\;\text{and}\;\left|\int_{\Omega }\varphi_{1}\varphi_{2}\;dx\right|\leq \frac{\epsilon }{2}∥\varphi_{1}{∥}_{L^{2}(\Omega )}^{2}+\frac{1}{2\epsilon }∥\varphi_{2}{∥}_{L^{2}(\Omega )}^{2},$$

that hold for all $\varphi_{1}\in L^{2}(\Omega )$ and $\varphi_{2}\in L^{2}(\Omega )$ and for any fixed ϵ∈(0,∞). In addition, we recall trace and Poincare's inequalities, [30],

(3) $$\begin{array}{rl} ∥\varphi {∥}_{L^{2}(\partial \Omega )}^{2} & \leq C_{tr}∥\varphi {∥}_{L^{2}(\Omega )}∥\varphi {∥}_{H^{1}(\Omega )},\\ ∥\varphi {∥}_{L^{2}(\Omega )} & \leq {meas}_{R^{d}}(\Omega )\;∥\nabla \varphi {∥}_{L^{2}(\Omega )},\;for\;\varphi \in H_{\Gamma }^{1}(\Omega ). \end{array}$$

### 2.2. The elliptic diffusion problem

The weak formulation of the boundary value problem (1) reads as follows: for given source function $f\in L^{2}(\Omega )$ find a function $u\in H_{0}^{1}(\Omega )$ such that the variational identity

(4) $$a(u,\varphi )=l_{f}(\varphi ),\;\forall \;\varphi \in H_{0}^{1}(\Omega ),$$

is satisfied, where the bilinear form a(⋅,⋅) and the linear form $l_{f}(\cdot )$ are defined by

(5) $$a(u,\varphi )=\int_{\Omega }\rho \nabla u\cdot \nabla \varphi \;dx\;\text{and}\;l_{f}(\varphi )=\int_{\Omega }f\varphi \;dx,$$

respectively. The given diffusion coefficient $\rho \in L^{\infty }(\Omega )$ is assumed to be uniformly positive and piece-wise (patch-wise, see below) constant. These assumptions ensure the existence and uniqueness of the solution due to Lax-Milgram's lemma. For simplicity, we only consider pure Dirichlet boundary conditions on ∂Ω. However, the analysis presented in our paper can easily be generalized to other constellations of boundary conditions that ensure existence and uniqueness such as Robin or mixed boundary conditions.

In what follows, positive constants *c* and *C* appearing in inequalities are generic constants that do not depend on the mesh-size *h*. In many cases, we will indicate on what may the constants depend on.

### 2.3. B-spline spaces

In this section, we briefly present the B-spline spaces and the form of the B-spline parametrizations for the physical subdomains. For a better presentation of the B-spline spaces, we start our discussion for the one-dimensional case. Then we proceed to higher dimensions. We refer to [2,4,31] for a more detailed presentation.

Consider, $Z=\{0=z_{1}<z_{2}<\cdots <z_{M}=1\}$ to be a partition of $\bar{I}=[0,1]$ with ${\bar{I}}_{j}=[z_{j},z_{j+1}],j=1,\ldots ,M-1$ to be the intervals of the partition. Let the integers *p* and $n_{1}$ denote the *p* spline degree and the number of the B-spline basis functions.

Based on Z, we introduce the open knot vector $\Xi =\{0=\xi_{1},\xi_{2},\ldots ,\xi_{n_{1}+p+1}=1\}$, and the associated vector $M=\{m_{1},\ldots ,m_{M}\}$ of knot multiplicities with $m_{1}=m_{M}=p+1$, i.e.

(6) $$\Xi =\{\underset{=z_{1}}{\underbrace{0=\xi_{1},\ldots ,\xi_{m_{1}}}},\underset{=z_{2}}{\underbrace{\xi_{m_{1}+1}=\ldots =\xi_{m_{1}+m_{2}}}},\ldots ,\underset{=z_{M}}{\underbrace{\xi_{n_{1}+p+1-m_{M}},\ldots ,\xi_{n_{1}+p+1}=1}}\}.$$

The B-spline basis functions are defined by the Cox-de Boor formula, see, e.g. [2,31],

(7) $$\begin{array}{rl} B_{i,p} & =\frac{x-\xi_{i}}{\xi_{i+p}-\xi_{i}}B_{i,p-1}(x)+\frac{\xi_{i+p+1}-x}{\xi_{i+p+1}-\xi_{i+1}}B_{i+1,p-1}(x),\\ with\;B_{i,0}(x) & =\left\{\begin{array}{ll} 1, & if\;\xi_{i}\leq x\leq \xi_{i+1},\\ 0, & otherwise \end{array}\right. \end{array}$$

We assume that $m_{j}\leq p$ for all internal knots, which in turn gives that, at $z_{j}$ the B-spline basis functions have $\kappa_{j}=p-m_{j}$ continuous derivatives.

Let us now consider the unit cube $\hat{\Omega }=(0,1{)}^{d}\subset R^{d}$, which we will refer to as the parametric domain. Let the integers *p* and $n_{k}$ denote the given B-spline degree and the number of basis functions of the B-spline space that will be constructed in $x_{k}$-direction with k=1,…,d. We introduce the *d*-dimensional vector of knots $\Xi =(\Xi^{1},\ldots ,\Xi^{k},\ldots ,\Xi^{d}),$ with the particular components given by $\Xi^{k}=\{0=\xi_{1}^{k},\xi_{2}^{k},\ldots ,\xi_{n_{k}+p+1}^{k}=1\}$, k=1,…,d,.

Given the knot vector $\Xi^{k}$ in every direction k=1,…,d and using (7), we construct the associated univariate B-spline basis functions, $\left\{{\hat{B}}_{1,k}({\hat{x}}_{k}),\ldots ,{\hat{B}}_{n_{k},k}({\hat{x}}_{k})\right\}$ of the space ${\hat{B}}_{\Xi^{k},p}$,

see, e.g. [31] for more details. Accordingly, the tensor product B-spline space is defined

(8) $${\hat{B}}_{\Xi ,p}={⊗}_{k=1}^{d}{\hat{B}}_{\Xi^{k},p}=span\{{\hat{B}}_{j}(\hat{x}){\}}_{j=1}^{n=n_{1}\cdot \ldots \cdot n_{k}\cdot \ldots \cdot n_{d}},$$

where each ${\hat{B}}_{j}(\hat{x})$ has the form

(9) $${\hat{B}}_{j}(\hat{x})={\hat{B}}_{j_{1}}({\hat{x}}_{1})\cdot \ldots \cdot {\hat{B}}_{j_{k}}({\hat{x}}_{k})\cdot \ldots \cdot {\hat{B}}_{j_{d}}({\hat{x}}_{d}),\;with\;{\hat{B}}_{j_{k}}({\hat{x}}_{k})\in {\hat{B}}_{\Xi^{k},p}.$$

In the IGA framework, the computational domain Ω is described as the image of $\hat{\Omega }$ under a B-spline parametrization mapping of the form

(10) $$\Phi :\hat{\Omega }\to \Omega ,\;x=\Phi (\hat{x})=\sum_{j=1}^{n}C_{j}{\hat{B}}_{j}(\hat{x})\in \Omega ,$$

where $C_{j},\;j=1,\ldots ,n$ are the control points and $\hat{x}=\Phi^{-1}(x)$, see Figure 1(a). Following the IGA methodology, [1,2], the B-spline spaces for discretizing the PDE problem are defined by using the mapping given in (10), i. e. we define the B-spline space in Ω by

(11) $$B_{\Xi ,p}:=span\{B_{j}{|}_{\Omega }:B_{j}(x)={\hat{B}}_{j}\circ \Phi^{-1}(x),\;for\;{\hat{B}}_{j}\in {\hat{B}}_{\Xi ,p}\}.$$

#### 2.3.1. B-spline spaces on multipatch representations

Let us suppose that the domain Ω can be described as a union of N-subdomains

(12) $$\bar{\Omega }=\overset{N}{\underset{i=1}{⋃}}{\bar{\Omega }}_{i},\;with\;\Omega_{i}\cap \Omega_{j}=\emptyset ,\;for\;i\neq j,$$

with interior interfaces $F_{ij}=\partial \Omega_{i}\cap \partial \Omega_{j}$, for 1≤i≠j≤N. We further suppose that every subdomain $\Omega_{i}$ has its own parametrization $\Phi_{i}$, which is defined by the corresponding B-spline space ${\hat{B}}_{\Xi_{i},p}$ and the corresponding control points $C_{j}^{\left(i\right)}$, see (10). Here $\Xi_{i}$ denotes the knot vector related to $\Omega_{i}$. An illustration for *N* = 2 is given in Figure 1(a). The subdomains $\Omega_{i}$ are referred to as patches. In an analogous way as in (11), we define the physical patch-wise B-spline spaces $B_{\Xi_{i},p}$ for i=1,…,N. We define the global discontinuous B-spline space $V_{B}$ with components on every $B_{\Xi_{i},p}$

(13) $$V_{B}:=\{\varphi_{h}\in L^{2}(\Omega ):\varphi_{h}{|}_{\Omega_{i}}\in B_{\Xi_{i},p}\}.$$

Assumption 2.1 Assume that every $\Phi_{i},\;i=1,\ldots ,N$ is sufficiently smooth and there exist constants 0<*c*<*C* such that $c\leq |\det J_{\Phi_{i}}|\leq C$, where $J_{\Phi_{i}}$ is the Jacobian matrix of $\Phi_{i}$.

The components of $\Xi_{i}$ form a mesh $T_{h_{i},\hat{\Omega }}^{\left(i\right)}=\{{\hat{E}}_{m}{\}}_{m=1}^{M_{i}}$ in $\hat{\Omega }$, where ${\hat{E}}_{m}$ are the micro-elements and $h_{i}$ is the mesh size, which is defined as follows. Given an element ${\hat{E}}_{m}\in T_{h_{i},\hat{\Omega }}^{\left(i\right)}$, we set $h_{{\hat{E}}_{m}}=diameter({\hat{E}}_{m})$ and the mesh size $h_{i}$ is defined to be $h_{i}=\max\{h_{{\hat{E}}_{m}}\}$. We set $h=\max_{i=1,\ldots ,N}\{h_{i}\}$. For every $\Omega_{i}$, we construct a mesh $T_{h_{i},\Omega_{i}}^{\left(i\right)}=\{E_{m}{\}}_{m=1}^{M_{i}}$, whose vertices are the images of the vertices of the corresponding parametric mesh $T_{h_{i},\hat{\Omega }}^{\left(i\right)}$ under $\Phi_{i}$.

Assumption 2.2 The meshes $T_{h_{i},\hat{\Omega }}^{\left(i\right)}$ are quasi-uniform, i.e. there exist a constant θ≥1 such that $\theta^{-1}\leq h_{{\hat{E}}_{m}}/h_{{\hat{E}}_{m+1}}\leq \theta$. Also, we assume that $h_{i}∼h_{j}$ for 1≤i≠j≤N.

### 2.4. Multipatch representation of the computational domain

In many practical applications, the parametrization of a boundary represented domain Ω by a single B-spline patch may not be possible. In order to discretize a PDE problem following the IGA framework in this situation, we represent the domain Ω as a multipatch. Following the methodology presented in [9,19], the initial domain Ω is firstly segmented into a collection of simple subdomains, e.g. topological hexahedra. Consequently, a suitable parametrization mapping is constructed for each subdomain for obtaining the multipatch representation of Ω. The final parametrization mappings of the adjoining patches must provide identical images for the common interfaces. In particular, for a DG-IGA discretization of the model (1), it would be preferable to produce a multipatch partition of Ω compatible with the variations of the coefficient *ρ*, i.e. the patches to be coincided with the parts of Ω where the coefficient *ρ* is constant. For example, let us consider Figure 1(a). In this case, the domain Ω is described as a union of two non overlapping patches, see (12), i.e.

(14) $$\bar{\Omega }=\bar{\Omega_{1}}\cup \bar{\Omega_{2}},\;\bar{\Omega_{1}}\cap \bar{\Omega_{2}}=\emptyset ,\;with\;F_{12}=\partial \Omega_{1}\cap \partial \Omega_{2},$$

where the interface $F_{12}$ coincides with the physical interface. We use the notation $T_{H}(\Omega ):=\{\Omega_{1},\Omega_{2}\}$ for the union (14). For each $\Omega_{i},\;i=1,2$, there exists a matching parametrization mapping such that $\Phi_{i}:\hat{\Omega }\to \Omega_{i}$ with $\Omega_{i}=\Phi_{i}(\hat{\Omega })$. The control points, which are related to the patch interface $F_{12}$, are appropriately matched in order for the parametrizations $\Phi_{1}$ and $\Phi_{2}$ of the neighboring patches to give the same image for the parametrized interface $F_{12}$. Based on $T_{H}(\Omega )$, we can independently discretize the problem on the different patches $\Omega_{i},\;i=1,2$, using interface conditions across $F_{12}$ for coupling the local problems. Typically, the interface conditions across $F_{12}$ concern continuity requirements of the solution *u* of (1), i.e.

(15) $$\text{⟦}u\text{⟧}:=u_{1}-u_{2}=0\;on\;F_{12},\;and\;\text{⟦}\rho \nabla u\text{⟧}\cdot n_{F_{12}}:=(\rho_{1}\nabla u_{1}-\rho_{2}\nabla u_{2})\cdot n_{F_{12}}=0\;on\;F_{i12},$$

where $n_{F_{12}}$ is the unit normal vector on $F_{12}$ with direction towards $\Omega_{2}$, and $\rho_{i},\;u_{i},\;i=1,2$ denote the restrictions of *ρ* and *u* to $\Omega_{i}$ correspondingly. The conditions (15) can be ensured by considering appropriate regularity assumptions on the solution *u*. We note that these types of multipatch representations have been considered in [6] and DG-IGA methods have been proposed for discretizing the problem (1).

Anyway, for simplicity, we develop our analysis based on Figure 1. We introduce the appropriate spaces. Let ℓ≥2 be an integer, we define the broken Sobolev space

(16) $$H^{\ell }(T_{H}(\Omega ))=\{u\in L^{2}(\Omega ):u_{i}=u{|}_{\Omega_{i}}\in H^{\ell }(\Omega_{i}),\;for\;i=1,2\}.$$

Assumption 2.3 We assume that the solution *u* of (4) belongs to $V=H_{0}^{1}(\Omega )\cap H^{2}(\Omega )\cap H^{\ell }(T_{H}(\Omega ))$ with ℓ≥2.

### 2.5. Problem statement

#### 2.5.1. Non-matching parametrized interfaces

**Figure 2.**
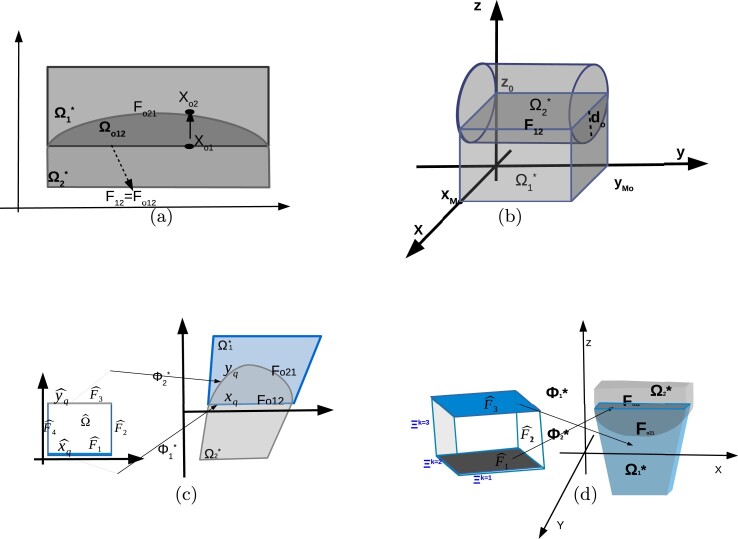
(a) Illustration of a patch representation with the overlapping region $\Omega_{o21}$ in 2d and the diametrically opposite points on $\partial \Omega_{o21}$, (b) overlapping patches in 3d, (c) the images of the faces of $\partial \hat{\Omega }$ under the mappings $\Phi_{i}^{*},\;i=1,2$ in 2d, (d) the images of the faces of $\partial \hat{\Omega }$ in 3d.

Typically, the segmentation procedure will generate multipatch representations that have possibly non-matching interface parametrizations, [10]. The result is the existence of gap and overlapping regions in the multipatch representation of the domain Ω. In [23,24], we developed DG-IGA schemes for multipatch unions that only include gap regions. In this work, we focus on multipatch representations with small overlapping regions, see Figures 1(b) and 2(a,b). Due to the non-matching parametrization of the interior patch interfaces, a direct application of the interface conditions (15) for deriving DG-IGA methods, is not possible. The purpose of this paper is to investigate the construction of auxiliary interface conditions on the boundary of the overlapping regions; which can be used for constructing DG-IGA schemes. We present a discretization error analysis separating the whole discretization error into two parts: the first naturally comes from the approximation properties of the B-spline spaces and the second, is the geometric error coming from the incorrect parametrization of the patches. The geometric error is considered as a consistency error and it is further separated into two components. The first error component is related to the approximation of the auxiliary flux terms across the non-matching interfaces and the second component is related to the existence of more than one numerical solution in the overlapping regions.

Remark 2.1 Alternatively, one can perform additional post-processing steps after the segmentation procedure to obtain matching interfaces. However, this procedure may increase the number of patches and the number of control points. Moreover, the newly obtained patch interfaces may not coincide with the original interface of the PDE problem, and thus the geometrical consistency error will still exist.

#### 2.5.2. The overlapping regions

As we mentioned above, for the sake of simplicity, we restrict our investigation to the case where the multipatch representation of Ω has two overlapping patches, see Figure 2. Let us suppose that

(17) $$\bar{\Omega }=\bar{\Omega_{1}^{*}}\cup \bar{\Omega_{2}^{*}},$$

where each patch has its own parametrization $\Phi_{1}^{*}:\hat{\Omega }\to \Omega_{1}^{*}$ and $\Phi_{2}^{*}:\hat{\Omega }\to \Omega_{2}^{*}$, as it is shown in Figure 2(c,d).

We denote the overlapping region by $\Omega_{o21}$, i.e. $\Omega_{o21}=\Omega_{1}^{*}\cap \Omega_{2}^{*}\subset \Omega$. We denote the interior boundary faces of the overlapping region by $F_{o12}=\partial \Omega_{1}^{*}\cap \Omega_{2}^{*}$ and $F_{o21}=\partial \Omega_{2}^{*}\cap \Omega_{1}^{*}$, which implies that $\partial \Omega_{o21}=F_{o12}\cup F_{o21}$. Finally, let $n_{F_{oij}}$ denote the unit exterior normal vector to $F_{oij}$, for 1≤i≠j≤2. For functions $u_{i}^{*}$ defined in $\Omega_{i}^{*},\;i=1,2$ we identify their pair $\left(u_{1}^{*},u_{2}^{*}\right)$ by $u^{*}$, which is equal to $u_{i}^{*}$ on $\Omega_{i}^{*}$. We develop our analysis for the case where the overlapping region is, at least locally, a convex region. We introduce an assumption related to the form of the faces $F_{o21}$ and $F_{o12}$. This assumption will help us to simplify the analysis, to explain our ideas in a better way, and to keep the notation to a minimum, e.g. the form of Jacobians, the form of face integrals, etc.

Assumption 2.4

- $\bar{\Omega_{1}}:=\bar{\Omega_{1}^{*}}$. The face $F_{o12}$ is an elementary face in the plane, i.e. is described by the points $\left\{(x,y,z):0\leq x\leq x_{M_{o}},\;0\leq y\leq y_{M_{o}},\;z=0\right\}$, and it coincides with the physical interface, i.e. $F_{o12}=F_{12}$, see (14).
- The face $F_{o21}$ can be described as the set of points (x,y,z) satisfying
  (18) $$0\leq x\leq x_{M_{o}},\;0\leq y\leq y_{M_{o}},\;z=\zeta_{0}(x,y),$$
  where $x_{Mo}$ and $y_{M_{o}}$ are real numbers, and $\zeta_{0}(x,y)$ is a given smooth function, see Figure 2.

We note that we will discretize the PDE problem using the B-spline spaces defined in $\Omega_{1}^{*}$ and $\Omega_{2}^{*}$, see (11). We will couple the resulting discrete problems in $\Omega_{1}^{*}$ and in $\Omega_{2}^{*}$ following discontinuous Galerkin techniques, this means by introducing appropriate numerical fluxes on $F_{o12}$ and on $F_{o21}$. In order to construct these fluxes, we assign the points located on $F_{o12}$ to the diametrically opposite points located on $F_{o21}$. Based on Assumption 2.4, we can construct a parametrization for the face $F_{o21}$, i.e. a mapping $\Phi_{o12}:F_{o12}\to F_{o21}$, of the form

(19) $$x_{o1}\in F_{o12}\to \Phi_{o12}(x_{o1}):=x_{o2}\in F_{o21},\;with\;\Phi_{o12}(x_{o1})=x_{o1}-\zeta_{o}(x_{o1})n_{F_{o12}},$$

where $n_{F_{o12}}$ is the unit normal vector on $F_{o12}$ and $\zeta_{o}$ has the same form as in (18), and it is a B-spline function with the same degree as the mapping $\Phi_{2}^{*}$, since the face $F_{o21}$ is the image of a face of $\partial \hat{\Omega }$ under the mapping $\Phi_{2}^{*}$. For the schematic illustration in Figure 2(c,d), we have $F_{o21}=\Phi_{2}^{*}({\hat{F}}_{3})$. Utilizing the mapping $\Phi_{o12}$ given in (19), we consider each point $x_{o2}\in F_{o21}$ as an image of a point $x_{o1}\in F_{o12}$ under the $\Phi_{o12}$, see Figure 2(a,c). Finally, we introduce a parameter $d_{o}$, which quantifies the width of the overlapping region $\Omega_{o21}$, i.e.

(20) $$d_{o}=\max_{x_{o1}\in F_{o12}}|x_{o1}-\Phi_{o12}(x_{o1})|.$$

In the present work, we are interested in overlapping regions with small size, and in particular for regions where their width $d_{o}$ decreases polynomially in *h*, i.e.

(21) $$d_{o}\leq h^{\lambda },\;with\;some\;\lambda \geq 1.$$

Based on this, we assume that $n_{F_{o12}}\approx -n_{F_{o21}}$, and define the mapping $\Phi_{o21}:F_{o21}\to F_{o12}$ as

(22) $$\Phi_{o21}(x_{o2})=x_{o1},\;with\;\Phi_{o12}(x_{o1})=x_{o2},$$

where $\Phi_{o21}$ is the inverse of $\Phi_{o12}$.

Remark 2.2 By introducing Assumption 2.4, the face $F_{o21}$ can be considered to be the graph of $\zeta_{0}$. This helps us to determine a parametrization for $F_{o21}$ using the function $\zeta_{0}$, and through this, we can relate to every point $x_{o2}\in F_{o21}$ a point $x_{o1}\in F_{o12}$. This has been achieved by the mapping $\Phi_{o12}$ in (19). The mapping $\Phi_{o12}$ here has a simple form and simplifies the analysis. Other mappings (even for more complex overlapping regions), for relating the points $x_{o2}\in F_{o21}$ to the points $x_{o1}\in F_{o12}$ can be constructed. For example, one can follow the ideas of minimum distance problem presented in [32] and can relate the points $x_{o2}\in F_{o21}$ to the points $x_{o1}\in F_{o12}$ by introducing the condition $(x_{o1}-x_{o2})//n_{F_{o12}}$. In any case, the parametrization mappings must satisfy the maximum width condition given in (21).

Remark 2.3 As we previously said, the face $F_{o12}$ is the image of a face of $\partial \hat{\Omega }$ under the mapping $\Phi_{1}^{*}$, for example in Figure 2(c) we have $F_{o12}=\Phi_{1}^{*}({\hat{F}}_{1})$. On the other hand, the face $F_{o21}$ is an interior curve for $\Omega_{1}^{*}$, see Figure 2(a,c). Thus, one could try to see $F_{o21}$ as an image of a curve ${\hat{F}}_{o21}\subset \hat{\Omega }$ under the mapping $\Phi_{1}^{*}$, i.e. $F_{o21}=\Phi_{1}^{*}({\hat{F}}_{o21})$. In that way, it would be advantageous to have a parametric description of $\partial \Omega_{o21}$ using the mapping $\Phi_{1}^{*}$, which in turn would help to link the diametrically opposite points $x_{o1}$ and $x_{o2}$, see (19). This approach requires the computation of the inverse $(\Phi_{1}^{*}{)}^{-1}$, which in general is very costly and demands the use of a Newton approach for solving many nonlinear systems. We are thus led to see the faces of $\partial \Omega_{o21}$ as images of both mappings $\Phi_{1}^{*}$ and $\Phi_{2}^{*}$. We note also that the mappings $\Phi_{o12}$ and $\Phi_{o21}$ are introduced and used only for deriving the discretization error analysis. They are not used in the computation of the entries of the system matrix of the discrete DG-IGA scheme, see also discussion in Subsection 4.1.

Remark 2.4 In Section 4, we give details of implementing the proposed method to more complicated overlapping regions. We present examples where $F_{o12}$ is not an elementary face in the plane and does not coincide with the physical phase $F_{12}$. Also, we note that for multipatch representations with large overlapping regions, one needs to work in a different direction and to use ideas coming from Schwarz domain-decomposition methods, see [33].

Remark 2.5 The methodology can also be applied to the case where the interior faces of $\partial \Omega_{o21}$ do not touch the boundary ∂Ω.

## 3. The patch-wise problems and the fluxes

We compute a numerical solution in each $\Omega_{i}^{*},\;i=1,2$ using the corresponding diffusion coefficient $\rho_{i}$ and the corresponding B-spline spaces defined in $\Omega_{i}^{*}$, lets us say $B_{\Xi_{i},p}^{*}$. Therefore on $\Omega_{o21}$ we will have the coexistence of two different numerical solutions and this makes the computation of the bounds for the error $∥u-u_{h}^{*}{∥}_{DG}$ more complicated. The norm $∥\cdot {∥}_{DG}$ is defined in (24). The idea in our approach is to introduce local (patch-wise) problems $a_{i}^{*}(u_{i}^{*},\varphi_{i}^{*})=l_{i,f}(\varphi_{i}^{*})$ in every $\Omega_{i}^{*}$, with appropriate bilinear forms $a_{i}^{*}(\cdot ,\cdot )$. Using the triangle inequality, we split the error as $∥u-u_{h}^{*}{∥}_{DG}\leq ∥u-u^{*}{∥}_{DG}+∥u^{*}-u_{h}^{*}{∥}_{DG}$. Then we estimate every term separately.

### 3.1. The patch-wise variational problems

Denote $T_{H}^{*}(\Omega ):=\{\Omega_{1}^{*},\Omega_{2}^{*}\}$, let ℓ≥1 be an integer and let the B-spline spaces $B_{\Xi_{i},p}^{*}$ defined in $\Omega_{i}^{*},\;i=1,2$. Accordingly to the spaces (16) and (13), we introduce the spaces

(23) $$\begin{array}{rl} H^{\ell }(T_{H}^{*}(\Omega )) & :=\{u^{*}=(u_{1}^{*},u_{2}^{*}):u_{i}^{*}\in H^{\ell }(\Omega_{i}^{*}),\;u_{i}^{*}{|}_{\partial \Omega_{i}^{*}\cap \partial \Omega }=0,\;for\;i=1,2\},\\ H_{0}^{\ell }(T_{H}^{*}(\Omega )) & :=\{u^{*}=(u_{1}^{*},u_{2}^{*}):u_{i}^{*}\in H_{0}^{\ell }(\Omega_{i}^{*}),\;for\;i=1,2\},\\ V_{B}^{*} & :=\{\varphi_{h}^{*}=(\varphi_{1,h},\varphi_{2,h}):\varphi_{i,h}\in B_{\Xi_{i},p}^{*},\;for\;i=1,2\}. \end{array}$$

In order to proceed, we first define the DG-norm $∥\cdot {∥}_{DG}$ associated with $T_{H}^{*}(\Omega )$. For all $v\in V_{h}^{*}:=H^{\ell }(T_{H}^{*}(\Omega ))+V_{B}^{*}$,

(24) $$∥v{∥}_{DG}^{2}=\sum_{i=1}^{2}\left(\rho_{i}∥\nabla v_{i}{∥}_{L^{2}(\Omega_{i}^{*})}^{2}+\frac{\rho_{i}}{h}∥v_{i}{∥}_{L^{2}(\partial \Omega_{i}^{*}\cap \partial \Omega )}^{2}+\sum_{F_{oij}\subset \partial \Omega_{i}^{*}}\frac{\left\{\rho \right\}}{h}∥v_{i}{∥}_{L^{2}(F_{oij})}^{2}\right),\;for\;1\leq i\neq j\leq 2,$$

where $F_{oij}$ are the interior faces related to overlapping regions, see Figure 2(a), and $\left\{\rho \}=\frac{1}{2}(\rho_{i}+\rho_{j}\right)$.

We recall Assumption 2.4 and the Dirichlet boundary conditions given in (1). On each $\Omega_{i}^{*},\;i=1,2$, we consider the auxiliary problems:

(25a) $$\begin{array}{rl} & \left\{\begin{array}{ll} -div(\rho_{1}\nabla u_{1}^{*})=f, & in\;\Omega_{1}^{*},\\ u_{1}^{*}=u_{D} & on\;\partial \Omega_{1}^{*}\cap \partial \Omega ,\\ u_{1}^{*}=u & on\;F_{o12}, \end{array}\right. \end{array}$$

(25b) $$\begin{array}{rl} & \left\{\begin{array}{ll} -div(\rho_{2}\nabla u_{2}^{*})=f, & in\;\Omega_{2}^{*},\\ u_{2}^{*}=u_{D} & on\;\partial \Omega_{2}^{*}\cap \partial \Omega ,\\ u_{2}^{*}=u & on\;F_{o21}, \end{array}\right. \end{array}$$

and furthermore, we consider the corresponding variational problems,

(26a) $$\begin{array}{rl} & find\;u_{1}^{*}\in H_{\partial \Omega_{1}^{*}\cap \partial \Omega }^{1}(\Omega_{1}^{*})\;such\;that\\ & \;u_{1}^{*}=u\;on\;F_{o12},\;and\;a_{1}^{*}(u_{1}^{*},\varphi_{1})=l_{1,f}^{*}(\varphi_{1}),\;for\;\varphi_{1}\in H_{0}^{1}(\Omega_{1}^{*}), \end{array}$$

where

(26b) $$\begin{array}{rl} & a_{1}^{*}(u_{1}^{*},\varphi_{1})=\int_{\Omega_{1}^{*}}\rho_{1}\nabla u_{1}^{*}\cdot \nabla \varphi_{1}\;dx,\;and\;l_{1,f}^{*}(\varphi_{1})=\int_{\Omega_{1}^{*}}f\varphi_{1}\;dx, \end{array}$$

(26c) $$\begin{array}{rl} & find\;u_{2}^{*}\in H_{\partial \Omega_{2}^{*}\cap \partial \Omega }^{1}(\Omega_{2}^{*})\;such\;that\\ & \;u_{2}^{*}=u\;on\;F_{o21},\;and\;a_{2}^{*}(u_{2}^{*},\varphi_{2})=l_{2,f}^{*}(\varphi_{2}),\;for\;\varphi_{2}\in H_{0}^{1}(\Omega_{2}^{*}), \end{array}$$

where

(26d) $$a_{2}^{*}(u_{2}^{*},\varphi_{2})=\int_{\Omega_{2}^{*}}\rho_{2}\nabla u_{2}^{*}\cdot \nabla \varphi_{2}\;dx,\;and\;l_{2,f}^{*}(\varphi_{2})=\int_{\Omega_{2}^{*}}f\varphi_{2}\;dx,$$

Remark 3.1 By Assumption 2.4 and the definition of problem (25a), we can imply that the solution *u* of (4) satisfies the problem (26a). The definition of (25b) and the fact that $\rho_{2}\neq \rho_{1}$ on $\Omega_{o21}$ imply that *u* of (4) does not satisfy the problem (26c).

According to Assumption 2.3, we make the following assumption.

Assumption 3.1 The solutions $u_{i}^{*},\;i=1,2$ in (26) belong to $H^{\ell }(T_{H}^{*}(\Omega ))$ with ℓ≥2.

In Appendix, see Subsection A.1, we give an estimate for the distance of the solutions *u* and $u^{*}$.

### 3.2. The non-consistent terms

We multiply (25b) by $\varphi_{2,h}\in B_{\Xi_{2},p}^{*}$, integrate over $\Omega_{2}^{*}$ and apply integration by parts, then after a few calculations we find that

(27) $$\begin{array}{rl} & \int_{\Omega_{2}^{*}}\rho_{2}\nabla u_{2}^{*}\cdot \nabla \varphi_{2,h}\;dx-\int_{\partial \Omega_{2}^{*}}\rho_{2}\nabla u_{2}^{*}\cdot n_{\partial \Omega_{2}^{*}}\varphi_{2,h}\;d\sigma \\ & \;=\int_{\Omega_{o21}}\rho_{1}\nabla u_{2}^{*}\cdot \nabla \varphi_{2,h}\;dx+\int_{\Omega_{2}}\rho_{2}\nabla u_{2}^{*}\cdot \nabla \varphi_{2,h}\;dx-\int_{\partial \Omega_{2}^{*}\cap \partial \Omega }\rho_{2}\nabla u_{2}^{*}\cdot n_{\partial \Omega_{2}}\varphi_{2,h}\;d\sigma \\ & \;-\int_{F_{o21}}\rho_{1}\nabla u_{2}^{*}\cdot n_{F_{o21}}\varphi_{2,h}\;d\sigma -\int_{F_{o12}}\rho_{1}\nabla u_{2}^{*}\cdot n_{F_{o12}}\varphi_{2,h}\;d\sigma -\int_{F_{o12}}\rho_{2}\nabla u_{2}^{*}\cdot (-n_{F_{o12}})\varphi_{2,h}\;d\sigma \\ & \;+\int_{\Omega_{o21}}(\rho_{2}-\rho_{1})\nabla u_{2}^{*}\cdot \nabla \varphi_{2,h}\;dx-\int_{F_{o12}}(\rho_{2}-\rho_{1})\nabla u_{2}^{*}\cdot n_{F_{o12}}\varphi_{2,h}\;d\sigma \\ & \;-\int_{F_{o21}}(\rho_{2}-\rho_{1})\nabla u_{2}^{*}\cdot n_{F_{o21}}\varphi_{2,h}\;d\sigma \\ & \;=l_{2,f}^{*}(\varphi_{2,h}). \end{array}$$

Working in a similar way, we multiply (25a) by $\varphi_{1,h}\in B_{\Xi_{1},p}^{*}$, and we have

(28) $$\int_{\Omega_{1}^{*}}\rho_{1}\nabla u_{1}^{*}\cdot \nabla \varphi_{h}\;dx-\int_{F_{o12}}\rho_{1}\nabla u_{1}^{*}\cdot n_{F_{o12}}\varphi_{h}\;d\sigma -\int_{\partial \Omega_{1}^{*}\cap \partial \Omega }\rho_{1}\nabla u_{1}^{*}\cdot n_{\partial \Omega_{1}}\varphi_{1,h}\;d\sigma =l_{1,f}^{*}(\varphi_{1,h}).$$

We define the forms

(29a) $$\begin{array}{rl} a_{2,h}^{*}(u_{2}^{*},\varphi_{2,h}) & :=\int_{\Omega_{2}^{*}}\;\rho_{2}\nabla u_{2}^{*}\cdot \nabla \varphi_{2,h}\;dx-\int_{F_{o21}}\;\rho_{2}\nabla u_{2}^{*}\cdot n_{F_{o21}}\varphi_{2,h}\;d\sigma -\int_{\partial \Omega_{2}^{*}\cap \partial \Omega }\;\rho_{2}\nabla u_{2}^{*}\cdot n_{\partial \Omega_{2}}\varphi_{2,h}\;d\sigma , \end{array}$$

(29b) $$\begin{array}{rl} a_{1,h}^{*}(u_{1}^{*},\varphi_{1,h}) & :=\int_{\Omega_{1}^{*}}\;\rho_{1}\nabla u_{1}^{*}\cdot \nabla \varphi_{1,h}\;dx-\int_{F_{o12}}\;\rho_{1}\nabla u_{1}^{*}\cdot n_{F_{o12}}\varphi_{1,h}\;d\sigma -\int_{\partial \Omega_{1}^{*}\cap \partial \Omega }\;\rho_{1}\nabla u_{1}^{*}\cdot n_{\partial \Omega_{1}}\varphi_{1,h}\;d\sigma \end{array}$$

as well as

(30a) $$\begin{array}{rl} a_{o,2}(u_{2}^{*},\varphi_{2,h}) & =\int_{\Omega_{o21}}\rho_{1}\nabla u_{2}^{*}\cdot \nabla \varphi_{2,h}\;dx+\int_{\Omega_{2}}\rho_{2}\nabla u_{2}^{*}\cdot \nabla \varphi_{2,h}\;dx-\int_{\partial \Omega_{2}^{*}\cap \partial \Omega }\rho_{2}\nabla u_{2}^{*}\cdot n_{\partial \Omega_{2}^{*}}\varphi_{2,h}\;d\sigma \\ & \;-\int_{F_{o21}}\rho_{1}\nabla u_{2}^{*}\cdot n_{F_{o21}}\varphi_{2,h}\;d\sigma -\int_{F_{o12}}\rho_{1}\nabla u_{2}^{*}\cdot n_{F_{o12}}\varphi_{2,h}\;d\sigma \\ & \;-\int_{F_{o12}}\rho_{2}\nabla u_{2}^{*}\cdot (-n_{F_{o12}})\varphi_{2,h}\;d\sigma \end{array}$$

(30b) $$\begin{array}{rl} a_{res}(u_{2}^{*},\varphi_{2,h}) & =\int_{\Omega_{o21}}(\rho_{2}-\rho_{1})\nabla u_{2}^{*}\cdot \nabla \varphi_{2,h}\;dx-\int_{F_{o12}}(\rho_{2}-\rho_{1})\nabla u_{2}^{*}\cdot n_{F_{o12}}\;\varphi_{2,h}\;d\sigma \\ & \;-\int_{F_{o21}}(\rho_{2}-\rho_{1})\nabla u_{2}^{*}\cdot n_{F_{o21}}\varphi_{2,h}\;d\sigma . \end{array}$$

By (27), (29) and (30), we get that

(31) $$a_{2,h}^{*}(u_{2}^{*},\varphi_{h})=a_{o,2}(u_{2}^{*},\varphi_{2,h})+a_{res}(u_{2}^{*},\varphi_{2,h})=l_{2,f}^{*}(\varphi_{2,h}),$$

Also for the solution *u* of (4) we have that

(32) $$\begin{array}{rl} & \int_{\Omega_{o21}}\rho_{1}\nabla u\cdot \nabla \varphi_{2,h}\;dx+\int_{\Omega_{2}}\rho_{2}\nabla u\cdot \nabla \varphi_{2,h}\;dx-\int_{F_{o21}}\rho_{1}\nabla u\cdot n_{F_{o21}}\varphi_{2,h}\;d\sigma \\ & \;-\int_{\partial \Omega_{2}^{*}\cap \partial \Omega }\rho_{2}\nabla u\cdot n_{\partial \Omega_{2}^{*}}\varphi_{1,h}\;d\sigma =l_{2,f}^{*}(\varphi_{2,h}). \end{array}$$

From the conditions (15), the forms defined in (29), (30) and the relations (31) and (32), we derive that

(33a) $$a_{o,2}(u_{2}^{*},\varphi_{2,h})+a_{res}(u_{2}^{*},\varphi_{2,h})=a_{o,2}(u,\varphi_{2,h})=l_{2,f}^{*}(\varphi_{2,h})$$

and

(33b) $$a_{2,h}^{*}(u,\varphi_{2,h})-a_{res}(u,\varphi_{2,h})=l_{2,f}^{*}(\varphi_{2,h}).$$

By a simple application of divergence theorem, we get

(34) $$a_{res}(u,\varphi_{2,h})=\int_{\Omega_{o21}}-div((\rho_{2}-\rho_{1})\nabla u)\varphi_{2,h}\;dx=\int_{\Omega_{o21}}\frac{\left(\rho_{2}-\rho_{1}\right)}{\rho_{1}}f\;\varphi_{2,h}\;dx.$$

Finally, by (33b) and (34), we deduce that

(35) $$a_{2,h}^{*}(u,\varphi_{2,h})+\int_{\Omega_{o21}}\frac{\left(\rho_{1}-\rho_{2}\right)}{\rho_{1}}f\;\varphi_{2,h}\;dx=l_{2,f}^{*}(\varphi_{2,h}).$$

Proposition 3.1 Let $\varphi_{2,h}\in B_{\Xi_{2},p}^{*}$. There is a *c*>0 dependent on *ρ* but independent of *u* and $\Omega_{o21}$ such that

(36) $$∥\varphi_{2,h}{∥}_{L^{2}(\Omega_{o21})}^{2}\leq cd_{o}\;h\left(\int_{\Omega_{2}^{*}}|\nabla \varphi_{2,h}{|}^{2}\;dx+\frac{\left\{\rho \right\}}{h}\int_{F_{o21}}\varphi_{2,h}^{2}\;d\sigma .\right)$$

Proof. Let $v=(0,y\varphi_{2,h}^{2})$. The divergence theorem for v on $\Omega_{o21}$ yields,

(37) $$\int_{\Omega_{o21}}\varphi_{2,h}^{2}\;dx+\int_{\Omega_{o21}}2y\varphi_{2,h}\;\partial_{y}\varphi_{2,h}\;dx=\int_{F_{o21}}y\varphi_{2,h}^{2}\;d\sigma .$$

Using that $y\leq d_{o}$ and applying (2) in (37) we obtain

(38) $$∥\varphi_{2,h}{∥}_{L^{2}(\Omega_{o21})}^{2}\leq \left(\epsilon^{2}\int_{\Omega_{o21}}\varphi_{2,h}^{2}\;dx+\frac{4}{\epsilon^{2}}\int_{\Omega_{o21}}d_{o}^{2}\;|\nabla \varphi_{2,h}{|}^{2}\;dx+d_{o}h\frac{1}{h}\int_{F_{o21}}\varphi_{2,h}^{2}\;d\sigma \right)$$

Gathering similar terms and choosing *ε* such that $1-\epsilon^{2}>0$, we get

(39) $$c_{1,\epsilon }∥\varphi_{2,h}{∥}_{L^{2}(\Omega_{o21})}^{2}\leq c_{2,\epsilon }c_{\rho }\;d_{o}h\left(\int_{\Omega_{2}^{*}}\rho_{2}|\nabla \varphi_{2,h}{|}^{2}\;dx+\frac{\left\{\rho \right\}}{h}\int_{F_{o21}}\varphi_{2,h}^{2}\;d\sigma \right),$$

where $c_{\rho }:=\max\{1/\rho_{2},1/\left\{\rho \right\}\}$ and we used that $d_{o}^{2}\leq d_{o}h$, see (21). Rearranging appropriately the constants in (39) yields (36).

Corollary 3.1 Let $f\in L^{\infty }(\Omega ),$ $\varphi_{2,h}\in B_{\Xi_{2},p}^{*}$ and let $u_{2}^{*}$ and *u* be the solutions of (26d) and (4) respectively. There are constants $c_{1},c_{\rho }>0$ dependent on $F_{o21}$ but independent of *h* such that

(40a) $$\begin{array}{rl} \int_{\Omega_{o21}}f\varphi_{2,h}\;dx & \leq c_{1}\;d_{o}\;∥f{∥}_{L^{\infty }(\Omega_{o21})}∥\varphi_{2,h}{∥}_{DG}, \end{array}$$

(40b) $$\begin{array}{rl} |a_{res}(u,\varphi_{2,h})| & \leq c_{\rho }\;d_{o}\;∥f{∥}_{L^{\infty }(\Omega_{o21})}∥\varphi_{2,h}{∥}_{DG}. \end{array}$$

Proof. The Cauchy–Schwartz inequality implies that

(41) $$\int_{\Omega_{o21}}f\varphi_{2,h}\;dx\leq ∥f{∥}_{L^{2}(\Omega_{o21})}∥\varphi_{2,h}{∥}_{L^{2}(\Omega_{o21})}\leq c_{F_{o}21}d_{o}^{1/2}∥f{∥}_{L^{\infty }(\Omega_{o21})}∥\varphi_{2,h}{∥}_{L^{2}(\Omega_{o21})}.$$

Using (36) in (41), the required assertion follows easily.

Inequality (40b) follows immediately from (34) and (40a).

### 3.3. The discrete problem

In this section, we use the bilinear forms given in (29) to define the patch-wise discrete problems. Based on Remark 3.1, and using the interface conditions on $F_{o21}$ and $F_{o12}$, which are introduced in (25a) and (25b), we imply the following interface condition

(42) $$u_{1}^{*}-u_{2}^{*}=0,\;on\;F_{o21}.$$

Next, we appropriately modify the flux terms $\int_{F_{o21}}\rho_{2}\nabla u_{2}^{*}\cdot n_{F_{o21}}\varphi_{2,h}\;d\sigma$ and $\int_{F_{o12}}\rho_{1}\nabla u_{1}^{*}\cdot n_{F_{o12}}\varphi_{1,h}\;d\sigma$ in (29) using Taylor expansions.

#### 3.3.1. Taylor expansions

Let $x,y\in {\bar{\Omega }}_{2}^{*}$ and let $f\in C^{m\geq 2}({\bar{\Omega }}_{2}^{*})$. We recall Taylor's formula with integral remainder

(43a) $$\begin{array}{rl} f(y) & =f(x)+\nabla f(x)\cdot (y-x)+R^{2}f(y+s(x-y)), \end{array}$$

(43b) $$\begin{array}{rl} f(x) & =f(y)-\nabla f(y)\cdot (y-x)+R^{2}f(x+s(y-x)), \end{array}$$

where $R^{2}f(y+s(x-y))$ and $R^{2}f(x+s(y-x))$ are the second order remainder terms defined by

(44a) $$\begin{array}{rl} R^{2}f(y+s(x-y)) & =\sum_{|\alpha |=2}(y-x{)}^{\alpha }\frac{2}{\alpha !}\int_{0}^{1}sD^{\alpha }f(y+s(x-y))\;ds, \end{array}$$

(44b) $$\begin{array}{rl} R^{2}f(x+s(y-x)) & =\sum_{|\alpha |=2}(x-y{)}^{\alpha }\frac{2}{\alpha !}\int_{0}^{1}sD^{\alpha }f(x+s(y-x))\;ds. \end{array}$$

#### Modifications of the fluxes on $\partial \Omega_{o21}$

3.3.2.

To illustrate the use of (43) in our analysis, we consider the simple case of Figure 2(a). Let the points $x_{o1}\in F_{o12}$ and $x_{o2}\in F_{o21}$ be such that $x_{o2}=\Phi_{o12}(x_{o1})$ as in Figure 2(a). We apply (43) using the points $x_{o1}$ and $x_{o2}$ and we have

(45a) $$f(x_{o1})-f(x_{o2})=\nabla f(x_{o2})\cdot (x_{o1}-x_{o2})+R^{2}f(x_{o1}+s(x_{o2}-x_{o1})),$$

and setting $x_{o2}=\Phi_{o12}(x_{o1})$ we have

(45b) $$f(x_{o1})-f(\Phi_{o12}(x_{o1}))=\nabla f(x_{o2}))\cdot (x_{o1}-x_{o2})+R^{2}f(x_{o1}+s(x_{o2}-x_{o1})).$$

In the same way we can get

(45c) $$f(x_{o2})-f(\Phi_{o21}(x_{o2}))=\nabla f(x_{o1}))\cdot (x_{o2}-x_{o1})+R^{2}f(x_{o2}+s(x_{o1}-x_{o2})).$$

Now denoting $r_{oij}=x_{oi}-x_{oj}$ we have that $n_{F_{oij}}=r_{oij}/|r_{oij}|.$ For keeping notation simple, we denote the Taylor's residuals as $R^{2}u_{x_{o1}}^{*}:=R^{2}u^{*}(x_{o1}+s(x_{o2}-x_{o1}))$ and $R^{2}u_{x_{o2}}^{*}:=R^{2}u^{*}(x_{o2}+s(x_{o1}-x_{o2}))$. Note that by the interface conditions (42), we have that $(\left\{\rho \right\}/h)\int_{F_{o21}}(u_{2}^{*}(x_{o2})-u_{1}^{*}(x_{o2}))\varphi_{2,h}\;d\sigma =0$, and using also (45) we modify the fluxes in (29) as follows

(46a) $$\begin{array}{rl} & \int_{F_{o21}}\rho_{2}\nabla u_{2}^{*}(x_{o2})\cdot n_{F_{o21}}\varphi_{2,h}\;d\sigma -\frac{\left\{\rho \right\}}{h}\int_{F_{o21}}(u_{2}^{*}(x_{o2})-u_{1}^{*}(x_{o2}))\varphi_{2,h}\;d\sigma \\ & \;=\int_{F_{o21}}\rho_{2}\nabla u_{2}^{*}(x_{o2})\cdot n_{F_{o21}}\varphi_{2,h}\;d\sigma -\frac{\left\{\rho \right\}}{h}\int_{F_{o21}}(u_{2}^{*}(x_{o2})-u_{1}^{*}(\Phi_{o21}(x_{o2}))\varphi_{2,h}\;d\sigma \\ & \;+\int_{F_{o21}}\frac{\left\{\rho \right\}}{h}(|r_{o21}|\nabla u_{2}^{*}(x_{o1})\cdot n_{F_{o21}}+R^{2}u_{x_{o2}}^{*})\varphi_{2,h}\;d\sigma , \end{array}$$

where $\left\{\rho \}=\frac{1}{2}(\rho_{1}+\rho_{2}\right)$. Similarly, we have

(46b) $$\begin{array}{rl} \int_{F_{o12}}\rho_{1}\nabla u_{1}^{*}(x_{o1})\cdot n_{F_{o12}}\varphi_{1,h}\;d\sigma & =\int_{F_{o12}}\rho_{1}\nabla u_{1}^{*}(x_{o1})\cdot n_{F_{o12}}\varphi_{1,h}\;d\sigma \\ & \;-\frac{\left\{\rho \right\}}{h}\int_{F_{o12}}(u_{1}^{*}(x_{o1})-u_{1}^{*}(x_{o1}))\varphi_{1,h}\;d\sigma \\ & =\int_{F_{o12}}\rho_{1}\nabla u_{1}^{*}(x_{o1})\cdot n_{F_{o12}}\varphi_{1,h}\;d\sigma \\ & \;-\frac{\left\{\rho \right\}}{h}\int_{F_{o12}}(u_{1}^{*}(x_{o1})-u_{1}^{*}(\Phi_{o12}(x_{o1}))))\varphi_{1,h}\;d\sigma \\ & \;+\frac{\left\{\rho \right\}}{h}\int_{F_{o12}}\nabla u_{1}(x_{o2})\cdot (x_{o1}-x_{o2})\\ & \;+R^{2}u_{1}(x_{o1}+s(x_{o2}-x_{o1})),\;(by\;(45a),\;(45b))\\ & =\int_{F_{o12}}\rho_{1}\nabla u_{1}^{*}(x_{o1})\cdot n_{F_{o12}}\varphi_{1,h}\;d\sigma \\ & \;-\frac{\left\{\rho \right\}}{h}\int_{F_{o12}}(u_{1}^{*}(x_{o1})-u_{2}^{*}(\Phi_{o12}(x_{o1})))\varphi_{1,h}\;d\sigma ,\\ & \;+\int_{F_{o12}}\frac{\left\{\rho \right\}}{h}(|r_{o12}|\nabla u_{1}^{*}(x_{o2})\cdot n_{F_{o12}}+R^{2}u_{x_{o1}}^{*})\varphi_{1,h}\;d\sigma ,\\ & \;(u_{2}^{*}(\Phi_{o12}(x_{o1}))=u_{1}^{*}(\Phi_{o12}(x_{o1})),\;see\;(42)). \end{array}$$

#### 3.3.3. The global modified form

We consider the global bilinear form $a^{*}(\cdot ,\cdot ):V_{h}^{*}\times V_{B}^{*}\to R$, which is formed by the contributions of $a_{i,h}^{*}(\cdot ,\cdot ),\;i=1,2$ given in (29) and the flux forms given in (46), that is

(47) $$\begin{array}{rl} a^{*}(u^{*},\varphi_{h}) & =a_{2,h}^{*}(u_{2}^{*},\varphi_{2,h})+a_{1,h}^{*}(u_{1}^{*},\varphi_{1,h})=\int_{\Omega_{1}^{*}}\rho_{1}\nabla u_{1}^{*}\cdot \nabla \varphi_{1,h}\;dx+\int_{\Omega_{2}^{*}}\rho_{2}\nabla u_{2}^{*}\cdot \nabla \varphi_{2,h}\;dx\\ & \;-\int_{\partial \Omega_{1}^{*}\cap \partial \Omega }\rho_{1}\nabla u_{1}^{*}\cdot n_{\partial \Omega_{1}^{*}}\varphi_{1,h}\;d\sigma -\int_{\partial \Omega_{2}^{*}\cap \partial \Omega }\rho_{2}\nabla u_{2}^{*}\cdot n_{\partial \Omega_{2}^{*}}\varphi_{2,h}\;d\sigma \\ & \;+\frac{\rho_{1}}{h}\int_{\partial \Omega_{1}^{*}\cap \partial \Omega }(u_{1}^{*}-u_{D})\varphi_{1,h}\;d\sigma +\frac{\rho_{2}}{h}\int_{\partial \Omega_{2}^{*}\cap \partial \Omega }(u_{2}^{*}-u_{D})\varphi_{2,h}\;d\sigma \\ & \;-\int_{F_{o12}}\left(\rho_{1}\nabla u_{1}^{*}(x_{o1})\cdot n_{F_{o12}}+\frac{\left\{\rho \right\}}{h}(u_{1}^{*}(x_{o1})-u_{2}^{*}(\Phi_{o12}(x_{o1}))\right)\varphi_{1,h}\;d\sigma \\ & \;-\int_{F_{o21}}\left(\rho_{2}\nabla u_{2}^{*}(x_{o2})\cdot n_{F_{o21}}+\frac{\left\{\rho \right\}}{h}(u_{2}^{*}(x_{o2})-u_{1}^{*}(\Phi_{o21}(x_{o2}))\right)\varphi_{2,h}\;d\sigma \\ & \;+\int_{F_{o21}}\frac{\left\{\rho \right\}}{h}(|r_{o21}|\nabla u_{2}^{*}(x_{o1})\cdot n_{F_{o21}}+R^{2}u_{x_{o2}}^{*})\varphi_{2,h}\;d\sigma \\ & \;-\int_{F_{o12}}\frac{\left\{\rho \right\}}{h}(|r_{o12}|\nabla u_{1}^{*}(x_{o2})\cdot n_{F_{o12}}+R^{2}u_{x_{o1}}^{*})\varphi_{1,h}\;d\sigma . \end{array}$$

Remark 3.2 Note that the exact solution *u* has similar regularity properties to the solution $u^{*}$, see Assumption 2.3, and thus we can derive for *u* an analogous formulation as this in (47).

#### 3.3.4. The DG-IGA scheme

In view of (47), we define the forms $A_{\Omega_{i}^{*}}(\cdot ,\cdot ):V_{h}^{*}\times V_{B}^{*}\to R$, $R_{\Omega_{o21}}(\cdot ,\cdot ):V_{h}^{*}\times V_{B}^{*}\to R$, and the linear functional $l_{f,\Omega_{i}^{*}}:V_{B}^{*}\to R$ by

(48a) $$\begin{array}{rl} A_{\Omega_{i}^{*}}(u^{*},\varphi_{h}) & =\sum_{i=1}^{2}(\int_{\Omega_{i}^{*}}\rho_{i}\nabla u_{i}^{*}\cdot \nabla \varphi_{i,h},\;dx-\int_{\partial \Omega_{i}^{*}\cap \partial \Omega }\rho_{i}\nabla u_{i}^{*}\cdot n_{\partial \Omega_{i}^{*}}\varphi_{i,h}\;d\sigma \\ & \;-\sum_{F_{oij}\subset \partial \Omega_{i}^{*}}\int_{F_{oij}}\rho_{i}\nabla u_{i}^{*}\cdot n_{F_{oij}}\varphi_{i,h}-\frac{\eta \{\rho \}}{h}(u_{i}^{*}-u_{j}^{*})\varphi_{i,h}\;d\sigma ),\;for\;1\leq i\neq j\leq 2, \end{array}$$

(48b) $$\begin{array}{rl} R_{\Omega_{o21}}(u^{*},\varphi_{h}) & =\int_{F_{o21}}\frac{\left\{\rho \right\}}{h}(|r_{o21}|\nabla u_{2}^{*}(x_{o1})\cdot n_{F_{o21}}+R^{2}u_{x_{o2}}^{*})\varphi_{2,h}\;d\sigma \\ & \;+\int_{F_{o12}}\frac{\left\{\rho \right\}}{h}(|r_{o12}|\nabla u_{1}^{*}(x_{o2})\cdot n_{F_{o12}}+R^{2}u_{x_{o1}}^{*})\varphi_{1,h}\;d\sigma ,\\ l_{f,\Omega_{i}^{*}}(\varphi_{h}) & =\sum_{i=1}^{2}\int_{\Omega_{i}^{*}}f\varphi_{i,h}\;dx, \end{array}$$

where η>0 is a parameter that is going to be determined later. Based on the forms defined in (48), we introduce the discrete bilinear form $A_{h}(\cdot ,\cdot ):V_{B}^{*}\times V_{B}^{*}\to R$ and the linear form $F_{h}:V_{B}^{*}\to R$ as follows

(49) $$\begin{array}{rl} A_{h}(u_{h}^{*},\varphi_{h}) & =A_{\Omega_{i}^{*}}(u_{h}^{*},\varphi_{h})+\sum_{i=1}^{2}\frac{\eta \rho_{i}}{h}\int_{\partial \Omega_{i}^{*}\cap \partial \Omega }u_{i,h}^{*}\varphi_{i,h}\;d\sigma , \end{array}$$

(50) $$\begin{array}{rl} F_{h}(\varphi_{h}) & =l_{f,\Omega_{i}^{*}}(\varphi_{h})+\sum_{i=1}^{2}\frac{\eta \rho_{i}}{h}\int_{\partial \Omega_{i}^{*}\cap \partial \Omega }u_{D}\varphi_{i,h}\;d\sigma . \end{array}$$

Finally, the DG-IGA scheme reads as follows: find $u_{h}^{*}\in V_{B}^{*}$ such that

(51) $$A_{h}(u_{h}^{*},\varphi_{h})=F_{h}(\varphi_{h}),\;for\;all\;\varphi_{h}\in V_{B}^{*}.$$

Remark 3.3 From the relations (31), (33), the Remark 3.2 and the forms given in (49) and in (50), we can derive that

(52) $$\begin{array}{rl} a_{2,h}^{*}(u_{2}^{*},\varphi_{2,h})+a_{1,h}^{*}(u_{1}^{*},\varphi_{1,h}) & =a_{o,2}(u_{2}^{*},\varphi_{2,h})+a_{res}(u_{2}^{*},\varphi_{2,h})+a_{1,h}^{*}(u_{1}^{*},\varphi_{1,h})\\ & =A_{h}(u^{*},\varphi_{h})+R_{\Omega_{o21}}(u^{*},\varphi_{h})\\ & =a_{o,2}(u,\varphi_{2,h})+a_{1,h}^{*}(u,\varphi_{1,h})\\ & =a_{2,h}(u,\varphi_{2,h})-a_{res}(u,\varphi_{2,h})+a_{1,h}^{*}(u,\varphi_{1,h})\\ & =A_{h}(u,\varphi_{h})+R_{\Omega_{o21}}(u,\varphi_{h})-a_{res}(u,\varphi_{2,h})\\ & =F_{h}(\varphi_{h}),for\;\varphi_{h}:=(\varphi_{1,h},\varphi_{2,h})\in V_{B}^{*}. \end{array}$$

Below, we quote few results that are useful for our error analysis. For the proofs we refer to [23–25].

Lemma 3.1 Under the assumption (21), there exist positive constants $C_{1}$ and $C_{2}$ independent of *h* such that the estimates

(53) $$|R_{\Omega_{o21}}(u,\varphi_{h})|\leq C_{1}K_{o}(u)∥\varphi_{h}{∥}_{DG}\;h^{\lambda -0.5},\;|R_{\Omega_{o21}}(u^{*},\varphi_{h})|\leq C_{2}K_{o}(u^{*})∥\varphi_{h}{∥}_{DG}\;h^{\lambda -0.5},$$

hold for the solutions $u^{*}$ and *u*, and $\varphi_{h}\in V_{B}^{*},$ where $K_{o}(v)=∥\nabla v{∥}_{L^{2}(\partial \Omega_{o21})}+∥\sum_{|\alpha |=2}|D^{\alpha }v|{∥}_{L^{2}(\Omega_{o21})}$.

Lemma 3.2 The bilinear form $A_{h}(\cdot ,\cdot )$ in (49) is bounded and elliptic on $V_{B}^{*},$ i.e. there are positive constants $C_{M}$ and $C_{m}$ such that the estimates

(54) $$A_{h}(v_{h},\varphi_{h})\leq C_{M}∥v_{h}{∥}_{DG}∥\varphi_{h}{∥}_{DG}\;and\;A_{h}(v_{h},v_{h})\geq C_{m}∥v_{h}{∥}_{DG}^{2},$$

hold for all $v_{h},\varphi_{h}\in V_{B}^{*}$ provided that *η* is sufficiently large, see [25].

Lemma 3.3 Let the assumption (21) and let $\text{β}=\lambda -\frac{1}{2}$. Then there is a constant $C_{*}>0$ depending on the parametrization mappings but independent of *h* such that the inequality

(55) $$A_{h}(v,\varphi_{h})\leq C_{*}{\left(∥v{∥}_{DG}^{2}+\sum_{i=1}^{2}h\;∥\rho_{i}^{1/2}\nabla v{∥}_{L^{2}(\partial \Omega_{i}^{*})}^{2}\right)}^{1/2}∥\varphi_{h}{∥}_{DG},$$

holds for all $(v,\varphi_{h})\in V_{h}^{*}\times V_{B}^{*}$ and $(v,\varphi_{h})\in (V+V_{B}^{*})\times V_{B}^{*}$.

Proof. Recall the definition of the pair function spaces in (23). In view of the form of $A_{h}(\cdot ,\cdot )$ and applying (2), we have

(56) $$\left|\sum_{i=1}^{2}\left(\int_{\Omega_{i}^{*}}\rho_{i}\nabla v_{i}\cdot \nabla \varphi_{i,h}\;dx)\right.\right|\leq {\left(\sum_{i=1}^{2}∥\rho_{i}^{1/2}\nabla v_{i}{∥}_{L^{2}(\Omega_{i}^{*})}^{2}\right)}^{1/2}{\left(\sum_{i=1}^{2}∥\rho_{i}^{1/2}\nabla \varphi_{i,h}{∥}_{L^{2}(\Omega_{i}^{*})}^{2}\right)}^{1/2}.$$

Now, let us first show an estimate for the normal fluxes on $F_{oij}$. Since $v\in V_{h}^{*}$ the normal traces on the interfaces are well defined. Using again (2), we obtain

(57) $$\begin{array}{rl} \left|\int_{F_{oij}}\rho_{i}\nabla v_{i}\cdot n_{F_{oij}}\varphi_{i,h}\;d\sigma \right| & \leq C_{i}\int_{F_{oij}}h^{1/2}|\rho_{i}^{1/2}\nabla v_{i}|{\left(\frac{\left\{\rho \right\}}{h}\right)}^{1/2}|\varphi_{i,h}|\;d\sigma \\ & \leq C_{i}\left(h^{1/2}∥\rho_{i}^{1/2}\nabla v_{i}{∥}_{L^{2}(F_{oij})}\right){\left(\frac{\eta \{\rho \}}{h}∥\varphi_{i,h}{∥}_{L^{2}(F_{oij})}^{2}\right)}^{1/2}\\ & \leq C_{i}\left(h^{1/2}∥\rho_{i}^{1/2}\nabla v_{i}{∥}_{L^{2}(F_{oij})}\right)∥\varphi_{h}{∥}_{DG}, \end{array}$$

for 1≤i≠j≤2. Also, we have

$$\begin{array}{rl} \left|\frac{\eta \{\rho \}}{h}\int_{F_{o12}}(v_{1}-v_{2}(\Phi_{o12}))\varphi_{1,h}\;d\sigma \right| & \leq 2{\left(\frac{\eta \{\rho \}}{h}\int_{F_{o12}}v_{1}^{2}+v_{2}^{2}(\Phi_{o12})\frac{|J_{\Phi_{o12}}|}{|J_{\Phi_{o12}}|}\;d\sigma \right)}^{1/2}\\ & \;\times {\left(\frac{\eta \{\rho \}}{h}∥\varphi_{1,h}{∥}_{L^{2}(F_{o12})}^{2}\right)}^{1/2}\\ & \leq C_{J_{\Phi_{o12}}}{\left(\frac{\eta \{\rho \}}{h}∥v_{1}{∥}_{L^{2}(F_{o12})}^{2}+\frac{\eta \{\rho \}}{h}∥v_{2}{∥}_{L^{2}(F_{o21})}^{2})\right)}^{1/2}\\ & \;\times {\left(\frac{\eta \{\rho \}}{h}∥\varphi_{1,h}{∥}_{L^{2}(F_{o12})}^{2}\right)}^{1/2}\\ & \leq C_{J_{\Phi_{o12}}}∥v{∥}_{DG}\;∥\varphi_{h}{∥}_{DG}, \end{array}$$

where $|J_{\Phi_{o12}}|$ is the measure of the Jacobian of $\Phi_{o12}$. In the same way, we show

$$\left|\frac{\eta \{\rho \}}{h}\int_{F_{o21}}(v_{2}-v_{1}(\Phi_{o21}))\varphi_{2,h}\;d\sigma \right|\leq C_{J_{\Phi_{o21}}}∥v{∥}_{DG}\;∥\varphi_{h}{∥}_{DG}.$$

Gathering together the above bounds, we show (55). For the case where $(v,\varphi_{h})\in (V+V_{B}^{*})\times V_{B}^{*}$ we work similarly.

### 3.4. Discretization error analysis

Next, we discuss interpolation estimates that we will use to bound the discretization error. We recall the definition of the pair function spaces in (23). Let $v\in H^{\ell }(T_{H}^{*}(\Omega ))$ with ℓ≥2. Under Assumptions 2.1, and using the results of [3,5], we can construct a quasi-interpolant $\Pi_{h}^{*}v:=(\Pi_{1,h}^{*}v_{1},(\Pi_{2,h}^{*}v_{2})\in V_{B}^{*}$ such that the estimates

(58) $$\begin{array}{rl} \sum_{i=1,2}|v-\Pi_{h}^{*}v{|}_{H^{1}(\Omega_{i}^{*})} & \leq h^{s}\sum_{i=1,2}C_{1,i}∥v{∥}_{H^{\ell }(\Omega_{i}^{*})},\\ \sum_{i=1,2}|v-\Pi_{h}^{*}v{|}_{L^{2}(\partial \Omega_{i}^{*})} & \leq h^{s-1/2}\sum_{i=1,2}C_{2,i}∥v{∥}_{H^{\ell }(\Omega_{i}^{*})}, \end{array}$$

hold, where s=min(ℓ−1,p) and the $C_{1,i}$, $C_{2,i}$ depend on $p,\Phi_{i}^{*},\theta$ but not on *h*.

Lemma 3.4 Let $v\in H^{\ell }(T_{H}^{*}(\Omega ))$ with ℓ≥2 and let $\Pi_{h}^{*}v$ be as in (58). Then there exist constants $C_{i}>0,$ i=1,2, depending on $p,\Phi_{i}^{*},\;i=1,2$ and the quasi-uniformity of the meshes but not on *h* such that

(59) $${\left(∥v-\Pi_{h}^{*}v{∥}_{DG}^{2}+\sum_{i=1}^{2}h∥\rho_{i}^{1/2}\nabla (v-\Pi_{h}^{*}v){∥}_{L^{2}(\partial \Omega_{i}^{*})}^{2}\right)}^{1/2}\leq \sum_{i=1}^{2}C_{i}h^{s}∥v{∥}_{H^{\ell }(\Omega_{i}^{*})},$$

where s=min(ℓ−1,p).

Proof. The estimate (59) can be shown using trace inequality and the estimates (58), see details in Lemma 10 in [6]. See also [23,24].

Theorem 3.1 Let $\text{β}=\lambda -\frac{1}{2}$ and $d_{o}=h^{\lambda }$ with λ≥1. Let $u^{*}\in H^{\ell }(T_{H}^{*}(\Omega ))$ with ℓ≥2 be the solution of the problems in (26), and let $u_{h}^{*}\in V_{B}^{*}$ be the corresponding DG-IGA solution of (51). Then the error estimate

(60) $$∥u^{*}-u_{h}^{*}{∥}_{DG}≲h^{r}\left(\sum_{i=1}^{2}∥u^{*}{∥}_{H^{\ell }(\Omega_{i}^{*})}\right),$$

holds, where r=min(s,β) with s=min(ℓ−1,p).

Proof. Let $z_{h}\in V_{B}^{*}$. We set $u_{h}^{*}-z_{h}=\varphi_{h}$. The properties (54), (55) of $A_{h}(\cdot ,\cdot )$ and (52) imply

(61) $$\begin{array}{rl} c_{m}∥u_{h}^{*}-z_{h}{∥}_{DG}^{2} & \leq A_{h}(u_{h}^{*}-z_{h},\varphi_{h})=A_{h}(u^{*},\varphi_{h})+R_{\Omega_{o21}}(u^{*},\varphi_{h})-A_{h}(z_{h},\varphi_{h})\\ & =A_{h}(u^{*}-z_{h},\varphi_{h})+R_{\Omega_{o21}}(u^{*},\varphi_{h})\\ & \leq C_{*}\left({\left(∥u^{*}-z_{h}{∥}_{DG}^{2}+\sum_{i=1}^{N}h\;∥\rho_{i}^{1/2}\nabla (u^{*}-z_{h}){∥}_{L^{2}(\partial \Omega_{i}^{*})}^{2}\right)}^{1/2}\right)∥\varphi_{h}{∥}_{DG}\\ & \;+C_{2}K_{o}(u^{*})∥\varphi_{h}{∥}_{DG}\;h^{\lambda -0.5}, \end{array}$$

where the bound (53) has been used previously. Setting in (61) $z_{h}=\Pi_{h}^{*}u^{*}$, and then using the triangle inequality $c_{m}∥u_{h}^{*}-u^{*}{∥}_{DG}\leq c_{m}∥u_{h}^{*}-\Pi_{h}^{*}u^{*}{∥}_{DG}+c_{m}∥u^{*}-\Pi_{h}^{*}u^{*}{∥}_{DG}$ together with the estimate in (59), we derive (60).

#### 3.4.1. Main error estimate

The estimate given in (60) concerns the distance between the DG-IGA solution $u_{h}^{*}\in V_{B}$ and the solution $u^{*}\in H^{\ell }(T_{H}^{*}(\Omega ))$ of the problems in (26). Below we give an estimate between the solution *u* of (4) and the DG-IGA solution $u_{h}^{*}$. In the proof of this result we need the following interpolation estimate for v∈V

(62) $${\left(∥v-\Pi_{h}^{*}v{∥}_{DG}^{2}+\sum_{i=1}^{2}h∥\rho_{i}^{1/2}\nabla (v-\Pi_{i,h}^{*}v){∥}_{L^{2}(\partial \Omega_{i}^{*})}^{2}\right)}^{1/2}\leq \sum_{i=1}^{2}C_{i}h^{s}∥v{∥}_{H^{\ell }(\Omega_{i}^{*})},$$

where the quasi-interpolant $\Pi_{h}^{*}v=(\Pi_{1,h}v,\Pi_{2,h}v)$ is defined in (58) and s=min(ℓ−1,p).

The proof of (62) is provided in the Appendix.

Theorem 3.2 **main error estimate**

Let *u* be the solution of (4) and let Assumption 2.3 with ℓ≥2. We suppose further that $d_{o}=h^{\lambda },\lambda \geq 1$ is the width of $\Omega_{o21}$. The following error estimate holds

(63) $$∥u-u_{h}^{*}{∥}_{DG}\leq \tilde{C}\left(h^{s}\sum_{i=1}^{2}(∥u{∥}_{H^{\ell }(\Omega_{i}^{*})}+∥u_{i}^{*}{∥}_{H^{\ell }(\Omega_{i}^{*})})+d_{o}∥f{∥}_{L^{2}(\Omega )}+h^{\text{β}}(K_{o}(u)+K_{o}(u^{*}))\right),$$

where $\text{β}=\lambda -\frac{1}{2},$ s=min(ℓ−1,p), the constant $\tilde{C}$ depends on the constants in (59), (55) and (54), and $K_{o}$ has the form given in Lemma 3.1.

Proof. Let $z_{h}\in V_{B}^{*}$ and let $\varphi_{h}=u_{h}^{*}-z_{h}$. By the definition of the discrete DG-IGA scheme in (51), the properties of $A_{h}(\cdot ,\cdot )$ and the Remark 3.3 we have

(64) $$\begin{array}{rl} c_{m}∥u_{h}^{*}-z_{h}{∥}_{DG}^{2} & \leq A_{h}(u_{h}^{*}-z_{h},\varphi_{h})-A_{h}(u^{*},\varphi_{h})-R_{\Omega_{o21}}(u^{*},\varphi_{h})+F_{h}(\varphi_{h})\\ & \;-A_{h}(\Pi_{h}^{*}u^{*},\varphi_{h})+A_{h}(\Pi_{h}^{*}u^{*},\varphi_{h})\\ & =A_{h}(u_{h}^{*}-\Pi_{h}^{*}u^{*},\varphi_{h})+A_{h}(u^{*}-\Pi_{h}^{*}u^{*},\varphi_{h})+A_{h}(-z_{h},\varphi_{h})+A_{h}(u,\varphi_{h})\\ & \;-a_{res}(u,\varphi_{2,h})+R_{\Omega_{o21}}(u,\varphi_{h})-R_{\Omega_{o21}}(u^{*},\varphi_{h})\\ & =A_{h}(u_{h}^{*}-\Pi_{h}^{*}u^{*},\varphi_{h})+A_{h}(u^{*}-\Pi_{h}^{*}u^{*},\varphi_{h})+A_{h}(u-z_{h},\varphi_{h})\\ & \;-a_{res}(u,\varphi_{2,h})+R_{\Omega_{o21}}(u,\varphi_{h})-R_{\Omega_{o21}}(u^{*},\varphi_{h})\\ & \leq C_{M}∥u_{h}^{*}-\Pi_{h}^{*}u^{*}{∥}_{DG}∥\varphi_{h}{∥}_{DG}\;by\;(53),(54),(55),(40)\\ & \;+C_{*}\left({\left(∥u^{*}-\Pi_{h}^{*}u^{*}{∥}_{DG}^{2}+\sum_{i=1}^{N}h\;∥\rho_{i}^{1/2}\nabla (u^{*}-\Pi_{h}^{*}u^{*}){∥}_{L^{2}(\partial \Omega_{i}^{*})}^{2}\right)}^{1/2}\right)∥\varphi_{h}{∥}_{DG}\\ & \;+C_{*}\left({\left(∥u-z_{h}{∥}_{DG}^{2}+\sum_{i=1}^{N}h\;∥\rho_{i}^{1/2}\nabla (u-z_{h}){∥}_{L^{2}(\partial \Omega_{i}^{*})}^{2}\right)}^{1/2}\right)∥\varphi_{h}{∥}_{DG}\\ & \;+c_{2}d_{o}∥f{∥}_{L^{2}(\Omega )}∥\varphi_{h}{∥}_{DG}+C_{2}(K_{o}(u^{*})+K_{o}(u))∥\varphi_{h}{∥}_{DG}\;h^{\text{β}} \end{array}$$

Setting $z_{h}=\Pi_{h}^{*}u$ into (64), using (61), (59), and (62) and gathering together the similar terms we deduce that

(65) $$\begin{array}{rl} c_{m}∥u_{h}^{*}-\Pi_{h}^{*}u{∥}_{DG} & \leq \sum_{i=1}^{2}C_{i}h^{s}∥u{∥}_{H^{\ell }(\Omega_{i}^{*})}+\sum_{i=1}^{2}C_{i}h^{s}∥u_{i}^{*}{∥}_{H^{\ell }(\Omega_{i}^{*})}\\ & \;+c_{2}d_{o}∥f{∥}_{L^{2}(\Omega )}+C_{2}(K_{o}(u^{*})+K_{o}(u))\;h^{\text{β}} \end{array}$$

Applying the triangle inequality

(66) $$∥u-u_{h}^{*}{∥}_{DG}\leq ∥u-\Pi_{h}^{*}u{∥}_{DG}+∥\Pi_{h}^{*}u-u_{h}^{*}{∥}_{DG},$$

the desired estimate follows.

Remark 3.4 In the description of the problem and in the derivation of the DG-IGA scheme, we focused on using B-spline spaces. The same derivation can be applied for the case of NURBS spaces.

## 4. Implementation and numerical tests

### 4.1. Implementation remarks

In this paragraph we focus on the implementation of the proposed scheme for both two and three dimensional problems. For simplicity of the presentation, we first discuss the case of having two patches. Afterwards, we explain how the same ideas can be generalized to the multipatch case.

**Figure 3.**
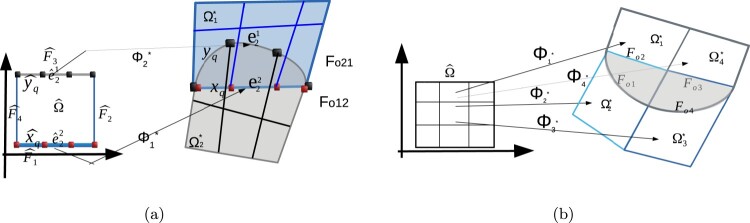
(a) Configuration of the faces and the edges on $\partial \Omega_{o12}$ and their corresponding edges on $\partial \hat{\Omega }$ which are used to compute the interface integrals, (b) an example of an overlapping region with more than two faces. The relative edges on the opposite faces must again match.

Initially, we consider interfaces with matching meshes, i.e. the number of edge elements on $F_{o21}$ is the same as the number on $F_{o12}$, as shown in Figure 3.

For the computation of the numerical flux terms of the DG-IGA scheme given in (48a), a Gauss quadrature rule is applied on every edge. The first term of the numerical flux can be directly computed by using the Gauss rule and the related Jacobian term. For the computation of the jump terms, we must know the diametrically opposite edge and the associated quadrature point that are located on the other interface. We could proceed to this direction by constructing and using the mappings $\Phi_{o21}$ and $\Phi_{o12}$ given in (19) and (22) respectively. For the practical implementation, it would be preferable to proceed without the construction of these mappings.

We first assign the edges belonging to $F_{o21}$ to the edges belonging to $F_{o12}$, for the example given in Figure 3(a), the edge $e_{2}^{1}$ of $F_{o21}$ is assigned to $e_{2}^{2}$ of $F_{o12}$. In Figure 3(a) the Gauss point is denoted by $x_{q}$ and $y_{q}$ correspondingly. The edge $e_{2}^{1}$ is the image of the edge ${\hat{e}}_{2}^{1}$ under the parametrization $\Phi_{2}^{*}$, and also the edge $e_{2}^{2}$ is the image of the edge ${\hat{e}}_{2}^{2}$ under the parametrization $\Phi_{1}^{*}$. Hence, the Gauss rule is transformed back to boundary edges of the parametric domain, and for every Gauss point ${\hat{y}}_{q}$ there is always a corresponding Gauss point on the other associated edge to perform the numerical integration. For the configuration given in Figure 3(a), the other associated edge is located on face ${\hat{F}}_{1}$ and the corresponding Gauss point is denoted by ${\hat{x}}_{q}$. Thus, having defined the quadrature points on the boundary edges of $\hat{\Omega }$, we can compute the interface terms of the numerical flux of the DG-IGA scheme.

Note that the above approach is quite simple and it follows the same ideas that we use for computing the numerical fluxes in the case of matching parametrized interfaces. It can be also applied for the case of having gap regions between the patches. The advantage of implementing this approach is that we can develop a flexible DG-IGA code which can treat patch unions with matching and non-matching interfaces in a similar way. Note also that the previous approach can be easily combined with the adaptive numerical quadrature methods presented in [34], in order to discretize the problem using non-matching structured meshes on the overlapping faces.

Overlapping regions with boundary consisting of more than two faces are shown in Figure 3(b). We consider again the case where the maximum number of the overlapping patches is two. For the example shown in Figure 3(b) the domain has four patches and the boundary of the overlapping region is compromised of the four faces $F_{oi},\;i=1,\ldots ,4$. Anyway, the evaluation of the interface numerical fluxes, in this case, needs more work. We first find the faces that form the boundary of the overlapping regions. Then between these faces, we determine those that are diametrically opposite, and we continue following the procedure described in the previous paragraph. This type of overlapping regions are discussed in the numerical Example 4.3.

It is clear that through a segmentation and parametrization procedure, overlapping regions with more complicated shapes than the shapes in the examples shown here can exist, e.g. more than two overlapping patches, T-joint faces on the boundary, see, e.g. [10]. In an ongoing work, we are extending the present methodology to treat these cases. We also are constructing domain-decomposition methods, [35], on these type of multipatch representations and we are discussing the influence of the size of the overlapping region on the performance of the proposed methods. We treat these cases by extending the ideas presented here. Again, we first find the interior faces that form the boundary of the overlapping regions and then we construct the numerical fluxes between the opposite faces in the same way as we presented in the previous sections. The first results of this work are included in [25]. We point out that when there are more than two overlapping patches, it is possible to have more than two (overlapped) numerical solutions on the overlapping regions. Consequently, in the error analysis, we will have more than one non-consistency terms to estimate, see Subsection 3.2. Also, we add that for multipatch unions with large overlapping regions, we can apply ideas coming from Schwarz domain-decomposition methods in order to treat the whole problem, see [29,33].

Finally, we mention that during the investigation of the proposed methodology in Section 3, we considered simple interior penalty fluxes on $\partial \Omega_{o21}$. For the performance of the numerical examples below, we have implemented the corresponding symmetric numerical fluxes, i.e.

$$-\int_{F_{o12}}\frac{1}{2}(\rho_{1}\nabla u_{1,h}+\rho_{2}\nabla u_{2,h}(\Phi_{o12}))\cdot n_{F_{o12}}\varphi_{1,h}+\frac{\eta \{\rho \}}{h}(u_{1,h}-u_{2,h}(\Phi_{o12}))\varphi_{1,h}\;ds.$$

See [6,23]. During the derivation of the discretization error analysis in [25], estimates and bounds for the values of the parameter *η* are introduced. Anyway for the numerical examples here we set $\eta =2(\left(p+1)(p+d\right)/d{)}^{1/2}$ where *d* is the dimension of Ω.

### 4.2. Numerical examples

In this section, we perform several numerical tests with different shapes of overlapping regions as well as combinations with non-homogeneous diffusion coefficients for two- and three-dimensional problems. We investigate the order of accuracy of the DG-IGA scheme proposed in (49). All examples have been performed using a second degree (*p* = 2) B-spline spaces. We present the asymptotic behavior of the error convergence rates for widths $d_{o}=h^{\lambda }$ with λ∈{1,2,2.5,3}. Every example has been solved applying several mesh refinement steps with $\ldots ,h_{i},h_{i+1},\ldots ,$ satisfying Assumption 2.2. The numerical convergence rates *r* have been computed by the ratio $r=\ln(e_{i}/e_{i+1})/\ln(h_{i}/h_{i+1}),\;i=1,2,\ldots$, where the error $e_{i}:=∥u-u_{h}^{*}{∥}_{DG}$ is always computed on the meshes $\overset{2}{\underset{i=1}{⋃}}T_{h_{i},\Omega_{i}^{*}}^{\left(i\right)}$. We mention that, in the test cases, we use highly smooth solutions in each patch, i.e. p+1≤ℓ, and therefore the order *s* in (60) and (63) becomes *s* = *p*. The predicted values of power *β*, the order *s* and the expected convergence rate *r*, for several values of *λ*, are displayed in Table 1. In any test case, the overlap regions are artificially created by moving the control points, which are related to the interfaces $F_{ij}$, in the direction of $n_{F_{ij}}$ or of $-n_{F_{ij}}$.

**Table 1.** The values of the expected rates *r* as they result from estimate (63).

	B-spline degree *p*			
	Smooth solutions, $u\in H^{\ell \geq p+1}$			
$d_{o}=h^{\lambda }$	λ=1	λ=2	λ=2.5	λ=3
β:=	0.5	1.5	2	2.5
s:=	*p*	*p*	*p*	*p*
r:=	0.5	1.5	min(p,β)	min(p,β)

All tests have been performed in G+SMO [36], which is a generic object-oriented C++ library for IGA computations, [37,38]. In Section 3, we developed and provided a rigorous analysis for the DG-IGA method (51) which includes a non-symmetric numerical flux. In the materialization of the method, we utilized the associated symmetrized version of the numerical flux, [39]. For solving the resulting linear system, we use the DG-IETI-DP method presented in [35], see also [40] for an analysis of the method and [41] for results on parallel scalability.

Although in the analysis, we consider meshes with similar quasi-uniform patch-wise properties, it is known that the introduction of DG techniques on the subdomain interfaces makes the use of non-matching and non-uniform meshes easier, see [6]. Keeping a constant linear relation between the sizes of the different patch meshes, the approximation properties of the method are not affected, [6]. In the examples below, we exploit this advantage of the DG methods and first solve two-dimensional problems considering non-matching meshes. The convergence rates are expected to be the same as those displayed in Table 1.

### 4.3. Two-dimensional numerical examples

The control points with the corresponding knot vectors of the domains given in Example 4.1–4.3 are available under the names yeti_mp2, 12pSquare and bumper as .xml files in G+SMO.^1^

Example 4.1 Uniform diffusion coefficient $\rho_{i}=1,\;i=1,\ldots ,N$

**Figure 4.**
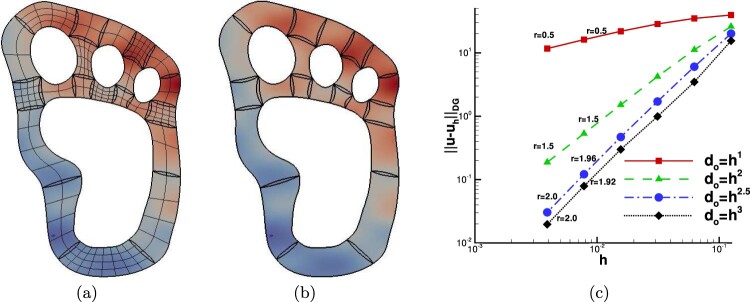
Example 4.1: (a) The patches $\Omega_{i}^{*}$ with the initial non-matching meshes and the contours of the exact solution. (b) The contours of the $u_{h}^{*}$ solution for $d_{o}=h$. (c) The convergence rates for the different values of *λ*.

The first numerical example is a simple test case demonstrating the applicability of the proposed technique for constructing the DG-IGA scheme on segmentations including overlaps with the general shape. The domain Ω with the *N* = 21 subdomains $\Omega_{i}^{*}$ and the initial mesh are shown in Figure 4(a). We note that we consider non-matching meshes across the interior interfaces. The Dirichlet boundary condition and the right-hand side *f* are determined by the exact solution u(x,y)=sin⁡(π(x+0.4)/6)sin⁡(π(y+0.3)/3)+x+y. In this example, we consider the homogeneous diffusion case, i.e. $\rho_{i}=1$ for all $\Omega_{i}^{*},\;i=1,\ldots ,N$.

We performed four groups of computations, where for every group the maximum size of $d_{o}$ was defined to be $O(h^{\lambda })$, with λ∈{1,2,2.5,3}. In Figure 4(b) we present the discrete solution for $d_{0}=h$. Since we are using second-order (*p* = 2) B-spline space, based on Table 1, we expect optimal convergence rates for λ=2.5 and λ=3. The numerical convergence rates for several levels of mesh refinement are plotted in Figure 4(c). They are in very good agreement with the theoretically predicted estimates given in Theorem 3.2, see also Table 1. We observe that we have optimal rates *r* for the cases where λ≥2.5 and suboptimal for the rest values of *λ*.

Example 4.2 Different diffusion coefficients $\rho_{1}\neq \rho_{2}$

**Figure 5.**
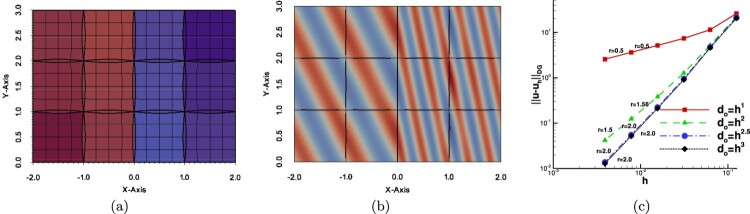
Example 4.2: (a) The overlapping patches $\Omega_{i}^{*}$ and the pattern of diffusion coefficients $\rho_{i}$, (b) The contours of $u_{h}^{*}$ on every $\Omega_{i}$ computed with $d_{0}=0.06$, (c) The convergence rates for the four choices of *λ*.

In the second example, we consider a rectangular domain Ω, that is described as a union of *N* = 12 patches, see Figure 5(a). Here, we study the case of having smooth solutions in each $\Omega_{i}^{*}$ but discontinuous coefficient, i.e. we set $\rho_{i}=3\pi /2$ for the patches belonging to half plane x≤0 and we set $\rho_{i}=2$ for the rest patches according to the pattern in Figure 5(a). By this example, we numerically validate the predicted convergence rates on $T_{H}^{*}$ with overlaps, for the case of having smooth solutions and discontinuous coefficient *ρ*. The exact solution is given by the formula

(67) $$u(x,y)=\left\{\begin{array}{ll} \sin(\pi (2x+y)) & ifx<0\\ \sin\left(\pi \left(\frac{3\pi }{2}x+y\right)\right) & otherwise. \end{array}\right.$$

The boundary conditions and the source function *f* are determined by (67). Note that, we have $\text{⟦}u\text{⟧}{|}_{F_{ij}}=0$ as well as $\text{⟦}\rho \nabla u\text{⟧}{|}_{F_{ij}}\cdot n_{F_{ij}}=0$ for all the interior physical interfaces $F_{ij}$.

The problem has been solved on a sequence of meshes with $h_{0},\ldots ,h_{i},\;h_{i+1},\ldots$, following a sequential refinement process, i.e. $h_{i+1}=h_{i}/2$, where we set $d_{o}=h_{i}^{\lambda }$, with λ∈{1,2,2.5,3}. For the numerical tests, we use B-splines of the degree *p* = 2. Hence, we expect optimal rates for λ≥2.5. In Figure 5(b) the approximate solution $u_{h}^{*}$ is presented on a relative coarse mesh with $d_{o}=0.06$. The results of the computed rates are presented in Figure 5(c). For all test cases, we can observe that our theoretical results presented in Table 1 are confirmed.

Example 4.3 **Overlapping regions with more than two faces**

**Figure 6.**
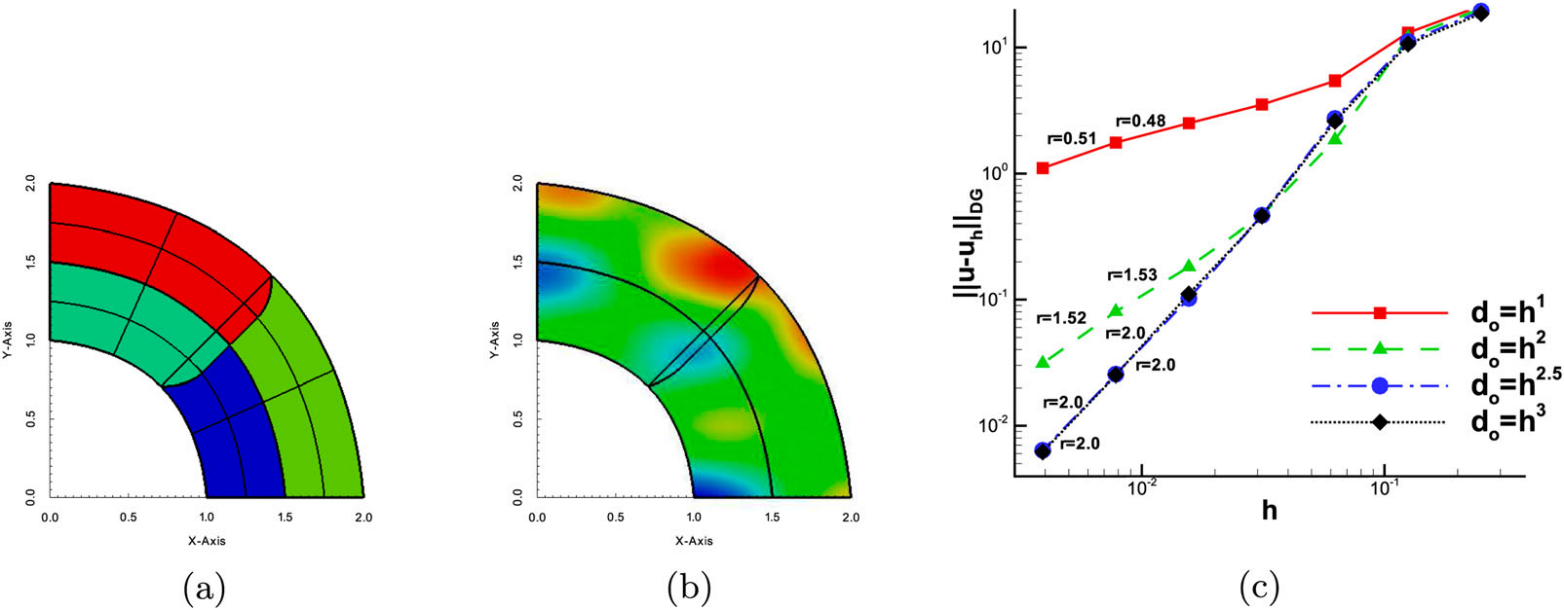
Example 4.3: (a) The overlapping patches $\Omega_{i}^{*}$ and the multiple curve boundary of the overlapping region, (b) The contours of $u_{h}^{*}$ on every $\Omega_{i}$ computed on the second mesh level, (c) The convergence rates for the four choices of *λ*.

The proposed method is now applied to a more complicated overlapping boundary with multiple faces. The geometric description of the problem in shown in Figure 6(a), the domain is decomposed into four patches and the overlapping region is defined by four interfaces. The exact solution is given by

(68) u(x,y)=sin⁡(π(x+0.4))sin⁡(2π(y+0.3))+x+y.

The diffusion coefficient is globally constant, i.e. ρ=1, the right-hand side *f* and the Dirichlet boundary conditions are manufactured by the solution (68). We solved the problem using B-splines of degree *p* = 2. In Figure 6(b), we present the contours of the DG-IGA solution $u_{h}^{*}$ computed on the second mesh in a sequence. The corresponding error convergence results for the four values of *λ*, i.e. λ∈{1,2,2.5,3}, are given in Figure 6(c). We can observe the suboptimal behavior of the rate for λ=1 and λ=2 as we move to the last mesh levels. On the other hand, we have optimal rates for the rest values of *λ*. The numerical rates for all *λ* cases are in agreement with the theoretical results.

### 4.4. Three-dimensional numerical examples

**Figure 7.**
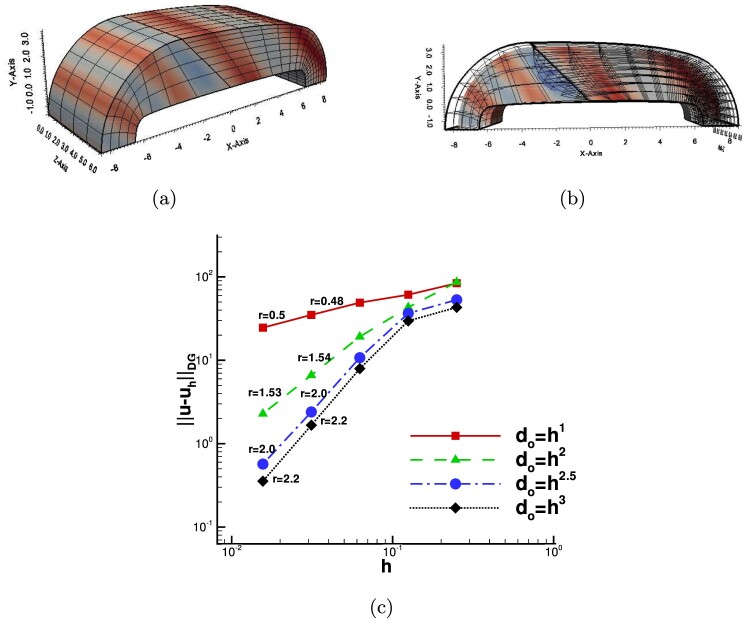
Example 4, $\Omega \subset R^{3}$: (a) The physical patches with an initial coarse mesh and the contours of the exact solution, (b) The contours of $u_{h}^{*}$ computed on $\Omega_{1}^{*}\cup \Omega_{2}^{*}$ with $d_{o}=1.5$, (c) Convergence rates *r* for the four values of *λ*.

As a final example, we consider a three-dimensional test. The domain Ω has been constructed by a straight prolongation to the *z*-direction of a two-dimensional (curved) domain, see Figure 7(a). The two physical domains $\Omega_{1}$ and $\Omega_{2}$ have the physical interface $F_{12}$ consisting of all points (x,y,z) such that −1≤x≤0,x+y=0 and 0≤z≤1, see Figure 7(a). The knot vector in the *z*-direction is simply $\Xi_{i}^{3}=\{0,0,0,0.5,1,1,1\}$ with *i* = 1, 2. We solve the problem using matching meshes, as depicted in Figure 7(a). The B-spline parametrizations of these domains are constructed by adding a third component to the control points with the following values {0,0.5,1}. The completed knot vectors $\Xi_{i=1,2}^{k=1,2,3}$ together with the associated control nets can be found in G+SMO library in the file bumper.xml. The overlap region is artificially constructed by moving only the interior control points located at the interface into the normal direction $n_{F_{12}}$ of the related interface $F_{12}$. Due to the fact that the overlap has to be inside the domain, we have to provide cuts through the domain in order to visualize them, cf. Figure 7(b). The Dirichlet boundary conditions $u_{D}$ and the right-hand side *f*, see (1), are chosen such that the exact solution is

(69) $$u(x,y,z)=\left\{\begin{array}{ll} \sin\left(\frac{\pi }{2}(x+y)\right) & if\;(x,y)\in \Omega_{1},\\ e^{\sin(x+y)} & if\;(x,y)\in \Omega_{2}. \end{array}\right.$$

with diffusion coefficient ρ={1,π/2}. Note that the interface conditions (15) are satisfied. The two physical subdomains, the initial matching meshes and the exact solution are illustrated in Figure 7(a). We construct an overlap region with $d_{o}=0.5$ and solve the problem using *p* = 2 B-spline functions. In Figure 7(b), we show the domain meshes $T_{h_{i},\Omega_{i}^{*}}^{\left(i\right)}\;,i=1,2$, the overlapped meshes in $\Omega_{o12}$ and we plot the contours of the produced solution $u_{h}^{*}$ for the interior plane *z* = 0.5. We can see that, both faces of $\partial \Omega_{o12}$ are not parallel to the Cartesian axes. Moreover, we point out that the problem has been solved using non-matching meshes on the overlapping interfaces. We have computed the convergence rates for four different values λ∈{1,2,2.5,3} related to the overlapping region width $d_{o}=h^{\lambda }$. The results of the computed rates are plotted in Figure 7(c). We observe from the plots that the rates *r* are in agreement with the rates predicted by the theory, see estimate (63) and Table 1.

## 5. Conclusions

In this article, we have proposed and analyzed a DG-IGA scheme for discretizing linear, second-order, diffusion problems on IGA multipatch representations with small overlapping regions. This type of multipatch representation leads to the use of different diffusion coefficients on the overlapping patches. Auxiliary problems were introduced in every patch and DG-IGA methodology applied for discretizing these problems. The normal fluxes on the overlapped interior faces were appropriately modified using Taylor expansions, and these fluxes were further used to construct numerical fluxes in order to couple the associated discrete DG-IGA problems. The method was successfully applied to the discretization of the diffusion problem in cases with complex overlaps. A priori error estimates in the DG-norm were shown in terms of the mesh-size *h* and the maximum width $d_{o}$ of the overlapping regions. The estimates were confirmed by solving several two- and three- dimensional test problems with known exact solutions. The theoretical estimates were also confirmed by performing numerical tests using non-matching grids on the overlapping faces.

## Note

1 G+SMO: https://www.gs.jku.at/trac/gismo

## Appendix

### A.1. A bound for the extra non-consistent term

Comparing the relations given in (27) and (33) we can see that there is an extra term $-(\rho_{2}-\rho_{1})\nabla u_{2}^{*}$ in $\Omega_{o21}$, which is a non consistent term. We derive below a bound for this term.

Let $\varphi \in H_{0}^{1}(\Omega_{2}^{*})$. By a simple computations on the forms in (26), we have that

(A1) $$\begin{array}{rl} a_{2}^{*}(u_{2}^{*},\varphi_{h}) & =\int_{\Omega_{o21}}\rho_{1}\nabla u_{2}^{*}\cdot \nabla \varphi \;dx+\int_{\Omega_{2}}\rho_{2}\nabla u_{2}^{*}\cdot \nabla \varphi \;dx-\int_{\partial \Omega_{2}\cap \partial \Omega }\rho_{2}\nabla u_{2}^{*}\cdot n_{\partial \Omega_{2}}\varphi \;d\sigma \\ & \;-\int_{F_{o21}}\rho_{2}\nabla u_{2}^{*}\cdot n_{F_{o21}}\varphi \;d\sigma =\int_{\Omega_{o21}}(\rho_{1}-\rho_{2})\nabla u_{2}^{*}\cdot \nabla \varphi \;dx+l_{2,f}^{*}(\varphi ). \end{array}$$

On the other hand, under the Assumption 2.3, we immediately have that

(A2) $$\begin{array}{rl} a_{o,2}(u,\varphi_{2}) & =\int_{\Omega_{o21}}\rho_{1}\nabla u\cdot \nabla \varphi \;dx+\int_{\Omega_{2}}\rho_{2}\nabla u\cdot \nabla \varphi \;dx\\ & \;-\int_{F_{o21}}\rho_{1}\nabla u\cdot n_{F_{o21}}\varphi \;d\sigma -\int_{\partial \Omega_{2}^{*}\cap \partial \Omega }\rho_{2}\nabla u\cdot n_{\partial \Omega_{2}}\varphi \;d\sigma =l_{2,f}^{*}(\varphi ). \end{array}$$

Subtracting (A2) from (A1) and using $\varphi {|}_{\partial \Omega_{2}^{*}}=0$ we obtain

(A3) $$\int_{\Omega_{o21}}\rho_{1}\nabla (u_{2}^{*}-u)\cdot \nabla \varphi \;dx+\int_{\Omega_{2}}\rho_{2}\nabla (u_{2}^{*}-u)\cdot \nabla \varphi \;dx=\int_{\Omega_{o21}}(\rho_{1}-\rho_{2})\nabla u_{2}^{*}\cdot \nabla \varphi \;dx.$$

Applying integration by parts on the right-hand side in (A3) and then setting $\varphi =u_{2}^{*}-u$, we derive that

(A4) $$\begin{array}{rl} & \int_{\Omega_{2}^{*}}\rho |\nabla (u_{2}^{*}-u){|}^{2}\;dx=c_{\rho }\left(-\int_{\Omega_{o21}}\rho_{2}\Delta u_{2}^{*}(u_{2}^{*}-u)\;dx+\int_{F_{o12}}\rho_{2}\nabla u_{2}^{*}\cdot n_{F_{o12}}(u_{2}^{*}-u)\;d\sigma \right)\\ & \;\leq c_{\rho }\left(\int_{\Omega_{o21}}f\;(u_{2}^{*}-u)\;dx+\int_{F_{o12}}\rho_{2}\nabla u_{2}^{*}\cdot n_{F_{o12}}(u_{2}^{*}-u)\;d\sigma \right)\\ & \;\overset{\left(2\right)}{\leq }c_{\rho }∥f{∥}_{L^{2}(\Omega_{o21})}∥u_{2}^{*}-u{∥}_{L^{2}(\Omega_{o21})}+∥\rho_{2}\nabla u_{2}^{*}{∥}_{L^{2}(F_{o12})}∥u_{2}^{*}-u{∥}_{L^{2}(F_{o12})}\\ & \;\overset{\left(3\right)}{\leq }c_{\rho }∥f{∥}_{L^{2}(\Omega_{o21})}\;∥u_{2}^{*}-u{∥}_{L^{2}(\Omega_{o21})}+∥\rho_{2}\nabla u_{2}^{*}{∥}_{L^{2}(F_{o12})}∥u_{2}^{*}-u{∥}_{L^{2}(\Omega_{o21})}^{1/2}∥u_{2}^{*}-u{∥}_{H^{1}(\Omega_{o21})}^{1/2}\\ & \;\overset{\left(3\right)}{\leq }c_{1}(∥f{∥}_{L^{2}(\Omega_{o21})}\;d_{o}∥\nabla (u_{2}^{*}-u){∥}_{L^{2}(\Omega_{o21})}+∥\rho_{2}\nabla u_{2}^{*}{∥}_{L^{2}(F_{o12})}\;d_{o}^{1/2}∥\nabla (u_{2}^{*}-u){∥}_{L^{2}(\Omega_{o21})}^{1/2}(d_{o}+1)∥\nabla (u_{2}^{*}-u){∥}_{L^{2}(\Omega_{o21})}^{1/2}\\ & \;\leq c_{2}(∥f{∥}_{L^{2}(\Omega_{o21})}+∥\rho_{2}\nabla u_{2}^{*}{∥}_{L^{2}(F_{o12})})\;d_{o}^{1/2}∥\nabla (u_{2}^{*}-u){∥}_{L^{2}(\Omega_{o21})}, \end{array}$$

where we have used that $0<d_{o}<1$. By (A4), we can easily obtain that

(A5) $$∥\rho \nabla (u_{2}^{*}-u){∥}_{L^{2}(\Omega_{2}^{*})}\leq c_{2}\;d_{o}^{1/2}(∥f{∥}_{L^{2}(\Omega_{o21})}+∥\rho_{2}\nabla u_{2}^{*}{∥}_{L^{2}(F_{o12})}),$$

and this gives an estimate of the difference between the physical solution *u* and the perturbed solution $u^{*}$.

### A.2. Proof of the interpolation estimate (62)

Note that by Assumption 2.4 and the definition of (25a) we can conclude that $\Omega_{1}=\Omega_{1}^{*}$ and $u{|}_{\Omega_{1}^{*}}=u_{\Omega_{1}^{*}}^{*}$. Hence we can construct an interpolant $\Pi_{1,h}^{*}u$ such that

(A6) $$\begin{array}{r} (\nabla (u_{1}-\Pi_{1,h}^{*}u_{1}){∥}_{L^{2}(\Omega_{1}^{*})}^{2}+h∥\nabla (u_{1}-\Pi_{1,h}^{*}u_{1}){∥}_{L^{2}(F_{o12})}^{2}+\frac{1}{h}∥(u_{1}-\Pi_{1,h}^{*}u_{1}){∥}_{L^{2}(F_{o21})}^{2}{)}^{1/2}\leq C_{1}h^{\min(\ell -1,p)}∥u{∥}_{H^{\ell }(\Omega_{1}^{*})}. \end{array}$$

Next we show an interpolation estimate for *u* on $\Omega_{2}^{*}$. Let us denote $D_{1}=\Omega_{o21}$ and $D_{2}=\Omega_{2}$. Let the extension operator $E_{i}:H^{\ell }(D_{i})\to H^{\ell }(\Omega_{i}^{*}),\;i=1,2,$ such that for each $v\in H^{\ell }(D_{i})$ it holds (i) $(E_{i}v){|}_{D_{i}}=v$ and (ii) $∥E_{i}v{∥}_{H^{\ell }(\Omega_{i}^{*})}\leq C_{E_{i}}∥v{∥}_{H^{\ell }(D_{i})}$, where the constant $C_{E_{i}}$ depending only on $D_{i}$ and $\Omega_{i}^{*}$, see [30]. We recall the B-spline interpolation operator$\Pi_{h}^{*}$ given in (58) and define

(A7) $$\Pi_{h}^{*}\tilde{v}:=(\Pi_{1,h}^{*}{\tilde{v}}_{1},\Pi_{2,h}^{*}{\tilde{v}}_{2}),\;where\;\Pi_{i,h}^{*}{\tilde{v}}_{i}:=\Pi_{i,h}^{*}E_{i}v.$$

Recalling $u_{i}=u{|}_{\Omega_{i}}$ and using the properties of the extension operator and (58) we have

(A8a) $$∥\nabla (u_{1}-\Pi_{1,h}^{*}{\tilde{u}}_{1}){∥}_{L^{2}(\Omega_{o21})}\leq ∥\nabla (E_{1}u-\Pi_{1,h}^{*}{\tilde{u}}_{1}){∥}_{L^{2}(\Omega_{1}^{*})}\leq C_{intp}C_{E_{1}}\;h^{s}∥u_{1}{∥}_{H^{\ell }(\Omega_{1}^{*})},$$

and

(A8b) $$∥\nabla (u_{2}-\Pi_{2,h}^{*}{\tilde{u}}_{2}){∥}_{L^{2}(\Omega_{2})}\leq ∥\nabla (E_{2}u-\Pi_{2,h}^{*}{\tilde{u}}_{2}){∥}_{L^{2}(\Omega_{2}^{*})}\leq C_{intp}C_{E_{2}}\;h^{s}∥u_{2}{∥}_{\Omega_{2}},$$

where s=min(p,ℓ−1).

Using the trace inequality, [6], $∥v{∥}_{L^{2}(F_{21})}^{2}\leq C(h^{-1}∥v{∥}_{L^{2}(\Omega_{o}21)}^{2}+h|\nabla v{∥}_{L^{2}(\Omega_{o21})}^{2})$ and proceeding as in (A8) we can show

(A9a) $$\begin{array}{rl} (h∥\nabla (u_{1}-\Pi_{1,h}^{*}{\tilde{u}}_{1}){∥}_{L^{2}(F_{o21})}^{2}{)}^{1/2} & \leq C_{intp}C_{E_{1}}\;h^{s}∥u_{1}{∥}_{H^{\ell }(\Omega_{1}^{*})}, \end{array}$$

(A9b) $$\begin{array}{rl} {\left(\frac{1}{h}∥(u_{1}-\Pi_{1,h}^{*}{\tilde{u}}_{1}){∥}_{L^{2}(F_{o21})}^{2}\right)}^{1/2} & \leq C_{intp}C_{E_{1}}\;h^{s}∥u_{1}{∥}_{H^{\ell }(\Omega_{1}^{*})}. \end{array}$$

Gathering the inequalities (A6), (A8) and (A9b) we can derive (62).

## References

[1] Hughes TJR, Cottrell JA, Bazilevs Y. Isogeometric analysis: CAD, finite elements, NURBS, exact geometry and mesh refinement. Comput Methods Appl Mech Eng. 2005;194:4135–4195. doi: 10.1016/j.cma.2004.10.008

[2] Cotrell JA, Hughes TJR, Bazilevs Y. Isogeometric analysis, toward integration of CAD and FEA. Sussex: John Wiley and Sons; 2009.

[3] da Veiga Beirão L, Buffa A, Sangalli G, et al. Mathematical analysis of variational isogeometric methods. Acta Numer. 2014 May;23:157–287. doi: 10.1017/S096249291400004X

[4] Schumaker LL. Spline functions: basic theory. Cambridge: University Press; 2007.

[5] Bazilevs Y, da Veiga Beirão L, Cottrell JA, et al. Isogeometric analysis: approximation, stability and error estimates for *h*-refined meshes. Math Models Meth Appl Sci. 2006;16(7):1031–1090. doi: 10.1142/S0218202506001455

[6] Langer U, Toulopoulos I. Analysis of multipatch discontinuous Galerkin IgA approximations to elliptic boundary value problems. Comput Vis Sci. 2016;17(5):217–233. doi: 10.1007/s00791-016-0262-6

[7] Tagliabue A, Dedé L, Quarteroni A. Isogeometric analysis and error estimates for high order partial differential equations in fluid dynamics. Comput Fluids. 2014;102:277–303. doi: 10.1016/j.compfluid.2014.07.002

[8] Hoschek J, Lasser D. Fundamentals of computet aided geometric design. Schumaker L, translator; Peters AK, editor. Wellesley (MA): AK Peters Ltd; 1993.

[9] Jüttler B, Kapl M, Nguyen D-M, et al. Isogeometric segmentation: the case of contractible solids without non-convex edges. Comput-Aided Des. 2014;57:74–90. doi: 10.1016/j.cad.2014.07.005

[10] Pauley M, Nguyen D-M, Mayer D, et al. The isogeometric segmentation pipeline. In: Jüttler B, Simeon B, editors. Isogeometric analysis and applications IGAA 2014. Heidelberg: Springer; 2015. (Lecture notes in computer science; vol. 107).

[11] Xu G, Mourrain B, Duvigneau R, et al. Analysis-suitable volume parameterization of multi-block computational domain in isogeometric applications. Comput-Aided Des. 2013;45(2):395–404. doi: 10.1016/j.cad.2012.10.022

[12] Xu G, Mourrain B, Duvigneau R, et al. Constructing analysis-suitable parameterization of computational domain from cad boundary by variational harmonic method. J Comput Phys. 2013;252(Supplement C):275–289. doi: 10.1016/j.jcp.2013.06.029

[13] Buchegger F, Jüttler B. Planar multi-patch domain parameterization via patch adjacency graphs. Comput-Aided Design. 2017;82:2–12. doi: 10.1016/j.cad.2016.05.019

[14] Falini A, Špeh J, Jüttler B. Planar domain parameterization with THB-splines. Comput Aided Geom Des. 2015;35–36:95–108. doi: 10.1016/j.cagd.2015.03.014

[15] Speleers H, Manni C. Optimizing domain parameterization in isogeometric analysis based on powell-sabin splines. J Comput Appl Math. 2015;289:68–86. doi: 10.1016/j.cam.2015.03.024

[16] Xu G, Li M, Mourrain B, et al. Constructing IgA-suitable planar parameterization from complex cad boundary by domain partition and global/local optimization. Comput Methods Appl Mech Eng. 2018;328(Supplement C):175–200. doi: 10.1016/j.cma.2017.08.052

[17] Engvall L, Evans JA. Isogeometric triangular Bernstein–Bézier discretizations: automatic mesh generation and geometrically exact finite element analysis. Comput Methods Appl Mech Eng. 2016;304:378–407. doi: 10.1016/j.cma.2016.02.012

[18] Xia S, Qian X. Generating high-quality high-order parameterization for isogeometric analysis on triangulations. Comput Methods Appl Mech Eng. 2018;338:1–26. doi: 10.1016/j.cma.2018.04.011

[19] Nguyen D-M, Pauley M, Jüttler B. Isogeometric segmentation. Part II: on the segmentability of contractible solids with non-convex edges. Graph Models. 2014;76:426–439. doi: 10.1016/j.gmod.2014.03.013

[20] Nguyen D-M, Pauley M, Jüttler B. Isogeometric segmentation: construction of auxiliarly curves. Comput-Aided Design. 2016;70:89–99. doi: 10.1016/j.cad.2015.06.014

[21] Bazilevs Y, Takizawa K, Tezduyar TE. Computational fluid – structure interaction, methods and applications. West Sussex: John Wiley and Sons, Ltd; 2013. (Wiley series in computational mechanics.).

[22] Nguyen VP, Kerfriden P, Brino M, et al. Nitsche's method for two and three dimensional NURBS patch coupling. Comput Mech. 2014;53(6):1163–1182. doi: 10.1007/s00466-013-0955-3

[23] Hofer C, Langer U, Toulopoulos I. Discontinuous Galerkin isogeometric analysis of elliptic diffusion problems on segmentations with gaps. SIAM J Sci Comput. 2016;38:A3430–A3460. doi: 10.1137/15M1048574

[24] Hofer C, Toulopoulos I. Discontinuous Galerkin isogeometric analysis of elliptic problems on segmentations with non-matching interfaces. Comput Math Appl. 2016;72(7):1811–1827. doi: 10.1016/j.camwa.2016.07.039

[25] Hofer C, Langer U, Toulopoulos I. Discontinuous Galerkin isogeometric analysis on non-matching segmentation: error estimates and efficient solvers. J Appl Math Comput. 2019;61:1–40. doi: 10.1007/s12190-019-01252-3

[26] Apostolatos A, Schmidt R, Wüchner R, et al. A Nitsche-type formulation and comparison of the most common domain decomposition methods in isogeometric analysis. Int J Numer Meth Eng. 2014;97:473–504. doi: 10.1002/nme.4568

[27] Bazilevs Y, Hughes TJR. Weak imposition of Dirichlet boundary conditions in fluid mechanics. Comput Fluids. 2007;36(1):12–26. doi: 10.1016/j.compfluid.2005.07.012

[28] Ruess M, Schillinger D, Özcan AI, et al. Weak coupling for isogeometric analysis of non-matching and trimmed multi-patch geometries. Comput Methods Appl Mech Eng. 2014;269(0):46–71. doi: 10.1016/j.cma.2013.10.009

[29] Zhang H, Mo R, Wan N. An IgA discontinuous Galerkin method on the union of overlapped patches. Comput Methods Appl Mech Eng. 2017;326:446–480. doi: 10.1016/j.cma.2017.08.004

[30] Evans LC. Partial differential equations. 1st ed. Providence: American Mathematical Society; 1998. (Graduate studies in mathematics; vol. 19).

[31] De-Boor C. A practical guide to splines. 2nd ed. New York: Springer; 2001. (Applied math. science; vol. 27).

[32] Wriggers P. Nonlinear finite element methods. Berlin: Springer-Verlag; 2008.

[33] Kargaran S, Jüttler B, Kleiss SK, et al. Overlapping multi-patch structures in isogeometric analysis. Linz: Johannes Kepler University, Applied Geometry; 2019. (NFN-technical report No. 87). Available from: http://www.gs.jku.at/pubs/NFNreport87.pdf.

[34] Seiler A, Jüttler B. Reparameterization and adaptive quadrature for the isogeometric discontinuous Galerkin method. 9th International Conference Mathematical Methods for Curves and Surfaces; MMCS 2016; Tønsberg, Norway, 2017. p. 251–269.

[35] Hofer C, Langer U. Dual-primal isogeometric tearing and interconnecting solvers for multipatch dG-IgA equations. Comput Methods Appl Mech Eng. 2017;316:2–21. doi: 10.1016/j.cma.2016.03.031

[36] Mantzaflaris A, Hofer C, Takacs S. G+SMO (Geometry plus simulation modules) v0.8.1; 2015. Available from: http://gs.jku.at/gismo

[37] Jüttler B, Langer U, Mantzaflaris A, et al. Geometry + simulation modules: implementing isogeometric analysis. PAMM. 2014;14(1):961–962. doi: 10.1002/pamm.201410461

[38] Langer U, Mantzaflaris A, Moore St. E, et al. Multipatch discontinuous Galerkin Isogeometric analysis. Heidelberg: Springer International; 2015. p. 1–32. (Lecture notes in computational science and engineering; vol. 107).

[39] Riviere B. Discontinuous Galerkin methods for solving elliptic and parabolic equations. Philadelphia: SIAM; 2008. (Society for industrial and applied mathematics).

[40] Hofer C. Analysis of discontinuous Galerkin dual-primal isogeometric tearing and interconnecting methods. Math Models Methods Appl Sci. 2018;28(01):131–158. doi: 10.1142/S0218202518500045

[41] Hofer C. Parallelization of continuous and discontinuous Galerkin dual-primal isogeometric tearing and interconnecting methods. Comput Math Appl. 2017;74(7):1607–1625. doi: 10.1016/j.camwa.2017.06.051

